# Identification and developmental expression of the full complement of Cytochrome P450 genes in Zebrafish

**DOI:** 10.1186/1471-2164-11-643

**Published:** 2010-11-18

**Authors:** Jared V Goldstone, Andrew G McArthur, Akira Kubota, Juliano Zanette, Thiago Parente, Maria E Jönsson, David R Nelson, John J Stegeman

**Affiliations:** 1 Biology Department, Woods Hole Oceanographic Institution, Woods Hole, MA, USA; 2 Andrew McArthur Consulting, 11 Roanoke Road, Hamilton, Ontario, Canada; 3 Instituto de Ciências Biológicas, Universidade Federal do Rio Grande, Rio Grande, RS 96201-900, Brazil; 4 Instituto de Biofísica Carlos Chagas Filho, Universidade Federal do Rio de Janeiro, Rio de Janeiro, Brazil; 5 Department of Environmental Toxicology, Uppsala University, Uppsala, Sweden; 6 Department of Molecular Sciences, University of Tennessee, Memphis, TN, USA

## Abstract

**Background:**

Increasing use of zebrafish in drug discovery and mechanistic toxicology demands knowledge of cytochrome P450 (CYP) gene regulation and function. CYP enzymes catalyze oxidative transformation leading to activation or inactivation of many endogenous and exogenous chemicals, with consequences for normal physiology and disease processes. Many CYPs potentially have roles in developmental specification, and many chemicals that cause developmental abnormalities are substrates for CYPs. Here we identify and annotate the full suite of CYP genes in zebrafish, compare these to the human CYP gene complement, and determine the expression of CYP genes during normal development.

**Results:**

Zebrafish have a total of 94 CYP genes, distributed among 18 gene families found also in mammals. There are 32 genes in CYP families 5 to 51, most of which are direct orthologs of human CYPs that are involved in endogenous functions including synthesis or inactivation of regulatory molecules. The high degree of sequence similarity suggests conservation of enzyme activities for these CYPs, confirmed in reports for some steroidogenic enzymes (e.g. CYP19, aromatase; CYP11A, P450scc; CYP17, steroid 17a-hydroxylase), and the CYP26 retinoic acid hydroxylases. Complexity is much greater in gene families 1, 2, and 3, which include CYPs prominent in metabolism of drugs and pollutants, as well as of endogenous substrates. There are orthologous relationships for some CYP1 s and some CYP3 s between zebrafish and human. In contrast, zebrafish have 47 CYP2 genes, compared to 16 in human, with only two (CYP2R1 and CYP2U1) recognized as orthologous based on sequence. Analysis of shared synteny identified CYP2 gene clusters evolutionarily related to mammalian CYP2 s, as well as unique clusters.

**Conclusions:**

Transcript profiling by microarray and quantitative PCR revealed that the majority of zebrafish CYP genes are expressed in embryos, with waves of expression of different sets of genes over the course of development. Transcripts of some CYP occur also in oocytes. The results provide a foundation for the use of zebrafish as a model in toxicological, pharmacological and chemical disease research.

## Background

The cytochrome P450 (CYP) enzymes catalyze oxidative transformation leading to activation or inactivation of many endogenous and exogenous chemicals, with consequences for normal physiology and disease processes. Mammalian CYPs can be separated into two major groups: those with generally narrow substrate specificity that are involved primarily in synthesis, activation or inactivation of endogenous regulatory molecules, and those involved most heavily in the metabolism of xenobiotics, but which may act as well in the metabolism of endogenous compounds. Thus, CYP enzymes can determine the persistence and action of endogenous regulatory molecules as well as many drugs and other toxicants and carcinogens.

An area growing in importance is the role of CYPs in development, and in developmental toxicity of chemicals. Absolute embryo lethality upon knockout of the murine CYP oxidoreductase (Por) demonstrates the essential role of CYP enzymes in murine development [1]. Many CYPs potentially have roles in developmental specification. For example, it is well known that CYP26 enzymes regulate levels of retinoids governing pattern formation during development [2-4]. CYP isoforms that play essential roles in bile acid homeostasis, steroidogenesis, the vitamin D pathway, and the catabolism of many hormones may also have important roles in development [5-12].

Many chemicals that cause developmental abnormalities, including cardiovascular, neural, and connective tissue defects, are substrates for CYPs, and the oxidative biotransformation of such xenobiotics may determine the cellular and organ targets of those chemicals [12]. CYP enzymes that are prominent in xenobiotic metabolism could also function in producing morphogenic molecules or keeping regions free of them, creating temporal and spatial regions of morphogen action and supporting region-specific changes essential for successful development [12-14]. The roles and regulation of most xenobiotic metabolizing CYPs during development are unknown, impeding understanding of mechanisms of developmental toxicity. Many xenobiotic metabolizing CYP can be induced via transcription factors (aryl hydrocarbon receptor, AHR; pregnane-X-receptor, PXR, peroxisome proliferator activated receptors, PPARs; and others [15,16]). All CYPs can be targets of exogenous chemicals that disrupt or enhance their function, whether or not they are directly involved in xenobiotic metabolism.

The zebrafish has emerged as one of the most important vertebrate model species in embryology and developmental biology [17,18], due largely to rapid development coupled with the possibility for genetic analysis and manipulation. Multiple draft assemblies of the zebrafish genome have been produced by the Wellcome Trust Sanger Institute [19]. The near completion of the zebrafish genome has made it possible to address questions of CYP gene identity and expression during zebrafish development with substantial clarity and completeness.

Our objective was to identify and annotate the full suite of zebrafish CYP genes and examine their phylogenetic and shared syntenic relationships to human CYPs. We also examined CYP expression during early development in zebrafish. CYP expression in vertebrate development has not been completely mapped for any species; prior studies in mice examined developmental expression of 40 CYP genes [10,20], less than half of the mouse CYP complement [21]. Our analyses identify novel genes and clusters of CYP genes in zebrafish, and unanticipated waves of CYP expression during development.

## Results

**Figure 1:**
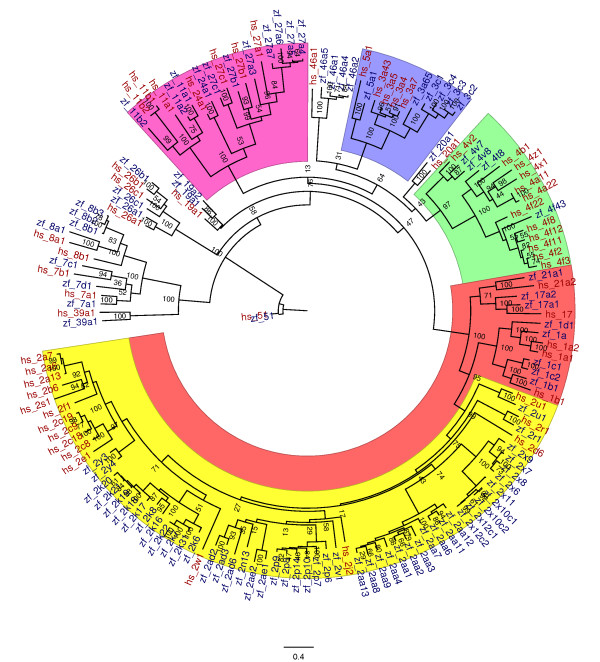
**Maximum likelihood phylogenetic tree of all zebrafish and human CYPs**. The phylogeny is rooted with CYP51, the only vertebrate CYP shared with fungi. Clans, as defined by Nelson (2003), are indicated by shaded highlights (red, Clan2; blue, Clan3; green, Clan4; purple, Mitochondrial Clan). The CYP2 family is further highlighted in yellow (see also Figure 2). Support values in the branches are derived from 100 bootstrap replicates using RAxML. Human (hs) sequences are red, zebrafish (zf) are blue.

Repeated and exhaustive searching of zebrafish genome assemblies uncovered a total of 94 CYP genes. Based on inferred amino acid sequences, these genes fell into 18 CYP gene families that are also found as well in humans and other mammals. Molecular phylogenetic analysis (Figure 1) shows relationships among CYP genes and gene families in zebrafish and human. As with the human CYP genes, the CYP genes in zebrafish occur in two major functional groups: those families that include enzymes involved primarily in endogenous functions (CYP families 5-51; Table 1); and those that include enzymes involved heavily in oxidation of xenobiotics (CYP families 1-3, and to a lesser degree family 4; Table 2). Zebrafish genes in these two major groups are described in detail below, considering relationship to human CYPs based on sequence identity, syntenic analysis and, where known, functional properties (functions of human or other mammalian homologs [22] are indicated in parentheses). The term CYP is fully capitalized in all cases except for mouse, rat, and *Drosophila *(where only the first letter is capitalized), and italicized only when referring to a specific gene or cDNA, consistent with the nomenclature committee recommendations [23].

**Table 1:** Zebrafish CYPs in families 5-51, with Chromosomal location (Zv8), Ensembl gene ID (Release 58), and exon count

Gene name	Ensembl 58 Gene ID	Ensembl 58 Transcript ID	Ensembl 58 Protein ID	Chromosome	Transcript Start (bp)	Transcript End (bp)	Strand	Exon	Ref
CYP5A1	ENSDARG00000002249	ENSDART00000109497	ENSDARP00000100741	18	12577084	12755668	1	13	

CYP7A1	ENSDARG00000069018	ENSDART00000100069	ENSDARP00000090841	2	20390259	20392545	-1	6	

CYP7C1	ENSDARG00000008858	ENSDART00000008268	ENSDARP00000020000	2	41226478	41238852	1	5	

CYP7D1	ENSDARG00000004051	ENSDART00000012822	ENSDARP00000007208	7	60786672	60803272	-1	6	

CYP8A1	ENSDARG00000060094	ENSDART00000084334	ENSDARP00000078769	6	50097042	50118485	-1	10	[26]

CYP8B1	ENSDARG00000053068	ENSDART00000053012^a^	ENSDARP00000091773	2	2631931	2633902	-1	1	

CYP8B2	ENSDARG00000059008	ENSDART00000053010^a^	listed as pseudogene	2	2598906	26000260	-1	1	

CYP8B3	ENSDARG00000059000	ENSDART00000012143	listed as pseudogene	2	2583060	2584972	-1	1	

CYP11A1	ENSDARG00000002347	ENSDART00000024866	ENSDARP00000025030	25	23348335	23363675	1	11	[117]

CYP11A2	ENSDARG00000051860	ENSDART00000073566	ENSDARP00000068056	25	23325076	23339692	1	9	

CYP11C1	ENSDARG00000042014	ENSDART00000061572	ENSDARP00000061571	16	51516891	51525686	1	10	

CYP17A1	ENSDARG00000033566	ENSDART00000043156	ENSDARP00000043155	13	33337340	33347115	1	8	[29]

CYP17A2	ENSDARG00000053966	ENSDART00000076086	ENSDARP00000070565	23	44130651	44138933	1	9	[32]

CYP19A1	ENSDARG00000041348	ENSDART00000060605	ENSDARP00000060604	18	39131244	39146992	-1	9	[34]

CYP19A2	ENSDARG00000009852	ENSDART00000025590	ENSDARP00000014320	25	3756544	3767668	1	10	[34]

CYP20A1	ENSDARG00000006040	ENSDART00000019325	ENSDARP00000003222	5	61726392	61743655	-1	15	

CYP21A1	ENSDARG00000037550	ENSDART00000059833^a^	ENSDARP00000059832	16	15091143	15104788	-1	12	

CYP24A1	ENSDARG00000070420	ENSDART00000103307^b^	ENSDARP00000094084	Zv8_NA3215	47572	49579	-1	12	

CYP26A1	ENSDARG00000033999	ENSDART00000041728	ENSDARP00000041727	12	9329895	9333488	-1	7	[41]

CYP26B1	ENSDARG00000077121	ENSDART00000110347	ENSDARP00000101543	7	25393384	25417689	1	6	[118]

CYP26C1 ^d^	ENSDARG00000056029	ENSDART00000077809	ENSDARP00000072275	17	17123846	17136305	1	7	[119]

CYP27A3	ENSDARG00000057262	ENSDART00000079879	ENSDARP00000074330	9	22363398	22377078	1	10	

CYP27A4	ENSDARG00000055159	ENSDART00000077479^c^	ENSDARP00000071946	9	39232071	39242971	1	9	

CYP27A5	ENSDARG00000055159	ENSDART00000108860^c^	ENSDARP00000101333	9	39213139	39219475	1	9	

CYP27A6	ENSDARG00000069186	ENSDART00000100487	ENSDARP00000091260	9	39205591	39211049	1	9	

CYP27A7	ENSDARG00000033802	ENSDART00000045588	ENSDARP00000045587	9	39194558	39204706	1	9	

CYP27B1	ENSDARG00000045015	ENSDART00000066178	ENSDARP00000066177	11	460256	466291	-1	11	

CYP27C1	ENSDARG00000058439	ENSDART00000081309	ENSDARP00000075752	6	10507141	10520886	-1	9	

CYP39A1	ENSDARG00000017982	ENSDART00000005764	ENSDARP00000016128	20	39084490	39112294	-1	12	

CYP46A1	ENSDARG00000012137	ENSDART00000028039	ENSDARP00000025234	20	4947206	4961574	1	15	

CYP46A2	ENSDARG00000004262	ENSDART00000057699	ENSDARP00000057698	5	73217357	73226060	-1	15	

CYP46A4	ENSDARG00000039492	ENSDART00000099258	ENSDARP00000090030	5	73235710	73241459	-1	15	

CYP46A5	not in	Ensembl	Build 58^e^						

CYP51A1	ENSDARG00000042641	ENSDART00000062551	ENSDARP00000062550	19	486980	505501	1	10	[54]

^a ^Incorrect predicted transcript; ^b ^incomplete best blast hit Chr6 58640777-58651921; ^c ^two different genes concatenated into one prediction; ^d ^originally published as CYP26D1; ^e ^Zv7 ENSDARG00000058474, Chromosome 20

**Table 2:** Zebrafish CYPs in families 1-4, with Chromosomal location (Zv8), Ensembl gene ID (Release 58), and exon count

Gene name	Ensembl 58 Gene ID	Ensembl 58 Transcript ID	Ensembl 58 Protein ID	Chromosome	Transcript Start (bp)	Transcript End (bp)	Strand	Exon	Ref
CYP1A	ENSDARG00000026039	ENSDART00000038200	ENSDARP00000033498	18	3972990	3983879	-1	7	[56]

CYP1B1	ENSDARG00000068934	ENSDART00000099870	ENSDARP00000090643	13	42451108	42458759	-1	2	[62]

CYP1C1	ENSDARG00000058980	ENSDART00000019953	ENSDARP00000004126	Zv8_scaf 3050	158017	161082	1	1	[62]

CYP1C2	ENSDARG00000018298	ENSDART00000016487	ENSDARP00000019538	Zv8_scaf 3050	152452	154638	1	1	[62]

CYP1D1	ENSDARG00000035569	ENSDART00000051565	ENSDARP00000051564	5	23578519	23593172	-1	7	[59]

CYP2AA1	ENSDARG00000070017	ENSDART00000102388	ENSDARP00000093164	23	39112545	39130047	1	9	

CYP2AA2	ENSDARG00000002981	ENSDART00000006065	ENSDARP00000021614^a^	23	39132514	39145108	1	9	

CYP2AA3	ENSDARG00000034070	ENSDART00000012284	ENSDARP00000014763	23	39160334	39172075	1	9	

CYP2AA4	ENSDARG00000002981	ENSDART00000045049	ENSDARP00000045048	23	39040573	39050888	1	9	

CYP2AA6	RNASEQDARG00000009292	RNASEQDART00000009292	N/A^b^	23	38994308	39009052	1	9	

CYP2AA7	ENSDARG00000002981	ENSDART00000007810	ENSDARP00000017742	23	39014695	39036855	1	9	

CYP2AA8	ENSDARG00000002981	ENSDART00000102420	ENSDARP00000093197	23	39075131	39083899	1	9	

CYP2AA9	ENSDARG00000070020	ENSDART00000020538	ENSDARP00000014406	23	39092849	39106493	1	9	

CYP2AA11	ENSDARG00000041418	ENSDART00000060715	ENSDARP00000060714	23	39025380	39036455	1	9	

CYP2AA12	ENSDARG00000002981	ENSDART00000102932	ENSDARP00000093707	23	38971320	38984900	1	9	

CYP2AA13		not in Ensembl Build 58							

CYP2AD2	ENSDARG00000021172	ENSDART00000024350	ENSDARP00000018819	20	25174055	25181567	-1	9	

CYP2AD3	ENSDARG00000022650	ENSDART00000063081	ENSDARP00000063080	20	25187810	25191060	-1	9	

CYP2AD6	ENSDARG00000042956	ENSDART00000063064	ENSDARP00000063063	20	25165262	25173123	-1	9	

CYP2AE1	ENSDARG00000013524	ENSDART00000035654	ENSDARP00000030778	23	39385086	39398666	1	10	

CYP2AE2	RNASEQDARG00000009297	RNASEQDART00000009297	NASEQDARP00000009294	23	39438355	39465937	1	10	

CYP2K6	ENSDARG00000038371	ENSDART00000055979	ENSDARP00000055978	3	8561027	8571051	-1	10	[75]

CYP2K7	ENSDARG00000040433	ENSDART00000101326	ENSDARP00000092100	3	6855362	6872308	-1	10	

CYP2K8	ENSDARG00000040431	ENSDART00000059182	ENSDARP00000059181	3	8585106	8602953	-1	9	

CYP2K16	ENSDARG00000058458	ENSDART00000081328	ENSDARP00000075771	3	8606661	8622465	-1	9	

CYP2K17	ENSDARG00000038369	ENSDART00000055971	ENSDARP00000055970	3	8624449	8631739	-1	9	

CYP2K18	ENSDARG00000038366	ENSDART00000055972	ENSDARP00000055971	3	8671991	8677064	-1	9	

CYP2K19	ENSDARG00000038367	ENSDART00000055973	ENSDARP00000055972	3	8647105	8654962	-1	9	

CYP2K20	ENSDARG00000040426	ENSDART00000059174	ENSDARP00000059173	3	8657079	8669242	-1	9	

CYP2K21	ENSDARG00000040424	ENSDART00000059171	ENSDARP00000059170	3	8638383	8643321	-1	9	

CYP2K22	ENSDARG00000040433	ENSDART00000015232	ENSDARP00000012186	3	6809065	6872358	-1	9	

CYP2K31	ENSDARG00000009874	ENSDART00000059185	ENSDARP00000059184	3	6887113	6898613	-1	9	

CYP2N13	ENSDARG00000042953	ENSDART00000063058	ENSDARP00000063057	20	25154644	25162165	-1	9	

CYP2P6^g^	ENSDARG00000042978	ENSDART00000063100	ENSDARP00000063099	20	25202489	25207822	1	9	[90]^g^

CYP2P7	ENSDARG00000042980	ENSDART00000063107	ENSDARP00000063106	20	25208167	25213192	1	9	

CYP2P8	ENSDARG00000042982	ENSDART00000063108	ENSDARP00000063107	20	25214780	25219066	1	9	

CYP2P9	ENSDARG00000022631	ENSDART00000030229	ENSDARP00000028831	20	25221011	25224248	1	9	

CYP2P10	ENSDARG00000042990	ENSDART00000063122	ENSDARP00000063121	20	25225641	25232985	1	9	

CYP2P14	ENSDARG00000042994	ENSDART00000063128	ENSDARP00000063127	20	25250397	25255115	1	8	

CYP2R1	ENSDARG00000056587	ENSDART00000079091	ENSDARP00000073546	7	27793618	27799151	1	5	

CYP2U1	ENSDARG00000026548	ENSDART00000048281	ENSDARP00000048280	1	49893260	49901816	-1	6	

CYP2V1	ENSDARG00000018485	ENSDART00000016501	ENSDARP00000011215	20	25191510	25197554	1	9	

CYP2X6	ENSDARG00000079653	ENSDART00000064591	ENSDARP00000064590	25	13917523	13924246	1	9	

CYP2X7	ENSDARG00000044002	ENSDART00000064586	ENSDARP00000064585	25	13906315	13912802	1	9	

CYP2X8	ENSDARG00000043997	ENSDART00000064596	ENSDARP00000064595	25	13889786	13898258	1	9	

CYP2X9	ENSDARG00000070775	ENSDART00000104216	ENSDARP00000094991	25	13881532	13888365	1	9	

CYP2X10c1	ENSDARG00000006501	ENSDART00000052054^c^	ENSDARP00000052053	7	52828535	52831744	-1	9	

CYP2X10c2	ENSDARG00000068283	ENSDART00000098690^c^	ENSDARP00000089461	7	52971112	52974318	-1	9	

CYP2X11	ENSDARG00000068287	ENSDART00000098703	ENSDARP00000089474	7	53000061	53012216	1	9	

CYP2X12c1	ENSDARG00000068290	ENSDART00000098705^d^	ENSDARP00000089476	7	53013611	53022923	1	9	

CYP2X12c2	ENSDARG00000068414	ENSDART00000098924^d^	ENSDARP00000089694	7	54082352	54244690	-1	10	

CYP2Y3	ENSDARG00000068493	ENSDART00000099074	ENSDARP00000089848	15	59576	64111	-1	9	

CYP2Y4	ENSDARG00000007173	ENSDART00000002842	ENSDARP00000013280	15	70518	76649	-1	9	

CYP3A65	ENSDARG00000045627	ENSDART00000067097	ENSDARP00000067096	1	59169705	59179378	-1	13	[80]

CYP3C1	ENSDARG00000015575	ENSDART00000018676	ENSDARP00000009507	3	38092409	38099696	-1	13	[79]

CYP3C2	ENSDARG00000037874	ENSDART00000055203	ENSDARP00000055202	3	38082058	38088687	-1	13	

CYP3C3	ENSDARG00000037873	ENSDART00000102393	ENSDARP00000093169	3	38071126	38078946	-1	13	

CYP3C4	ENSDARG00000070021	ENSDART00000102416	ENSDARP00000093193	3	38059261	38068888	-1	13	

CYP4F43	ENSDARG00000053530	ENSDART00000063442	ENSDARP00000063441	12	46414253	46432708	1	12	

CYP4T8	ENSDARG00000004964	ENSDART00000076955^e^	ENSDARP00000071423	16	3042131	3063539	-1	12	

CYP4V7	ENSDARG00000061585	ENSDART00000087976	ENSDARP00000082409	Zv8 NA9570^f^	11333	19450	1	11	

CYP4V8	ENSDARG00000062132	ENSDART00000089480	ENSDARP00000083913	1	17310768	17323080	1	11	

^a ^GeneWise reprediction; ^b ^no Ensembl gene prediction; ^c ^identical DNA; ^d ^identical DNA; ^e ^incorrect N-term exon; ^f ^Chr16 31205762 is probably best location; ^g ^There are non-mammalian genes that share synteny but not the same subfamily name with mammalian CYP2J2 s. At present, annotation of fish CYP2 s by BLAST can result in misclassifying fish CYP2 s as CYP2Js (Nelson, 2005, Wang et al. 2007), as strictly interpreted according to nomenclature rules, there is not a CYP2J subfamily in fish.

### CYP Families 5-51

#### CYP5 (thromboxane A2 synthase, TBXAS1

The mammalian CYP5A1 enzyme catalyzes rearrangement of prostaglandin H2 (PGH2). Zebrafish *CYP5A1 *retains 48% sequence identity with human *CYP5A1*, and is located in a region of shared synteny with *CYP5A1 *in humans, indicating orthology.

#### CYP7 (steroid 7α-hydroxylase)

Zebrafish possess one six-exon *CYP7A1 *and one six-exon *CYP7B1 *gene, both located on Chromosome 2. The sequence identity between zebrafish *CYP7B1 *and mammalian *CYP7Bs *technically precludes membership in the same subfamily, yet shared synteny (Table 3) indicates orthology. Zebrafish also have a third *CYP7 *gene, *CYP7C1*, a *CYP7A*-like gene located on Chromosome 7, possibly an ohnolog resulting from the fish-specific whole genome duplication (WGD 3; [24]).

**Table 3:** Synteny comparison between zebrafish and human CYPs

Zebrafish	Human
CYP1A	CYP1A1/1A2
CYP1B1	CYP1B1
CYP1C1,2	-
CYP1D1	CYP1D1P
CYP2Ks	CYP2W1
CYP2N13	CYP2J2
CYP2Ps	CYP2J2
CYP2R1	CYP2R1
CYP2U1	CYP2U1
CYP2V1	CYP2J2
CYP2X1-10	-
CYP2Y3,4	CYP2A/B/F/S
CYP2AA1-12	-
CYP2AD2,3,6	CYP2J2
CYP2AE1,2	-
CYP3A65	CYP3A-se1,-se2^a^
CYP3C1-4	CYP3A3,4,7
CYP4F43	CYP4F
CYP4V7,8	CYP4V2
CYP4T8	-
CYP5A1	CYP5A1
CYP7A1	CYP7A1
CYP7B1	CYP7B1
CYP7C1	-
CYP8A1	CYP8A1
CYP8B1-3	CYP8B1
CYP11A1,2	CYP11A1
CYP11C1	-
CYP17A1,2	CYP17A1
CYP19A1,2	CYP19A1
CYP20A1	CYP20A1
CYP21A1	CYP21A2
CYP24A1	CYP24A1
CYP26A1	CYP26A1/C1
CYP26B1	CYP26B1
CYP26C1	-
CYP27A3-7	CYP27A1
CYP27B1	-
CYP27C1	-
CYP39A1	CYP39A1
CYP46A1	CYP46A1
CYP46A2,4,5	-
CYP51A1	CYP51A1

^a ^pseudogene (single remnant exon)

#### CYP8 (prostacyclin synthase, PTGIS)

A 10-exon zebrafish *CYP8A1 *gene is located on chromosome 6. Human CYP8A1 catalyzes the rearrangement of PGH2 to prostaglandin I2 (prostacyclin) [25]. A crystal structure of zebrafish CYP8A1 published in 2008 as part of a comparative structural analysis of prostacyclin synthases, shows that zebrafish and human CYP8A1 exhibit nearly identical ligand-bound and ligand-free three-dimensional structures, despite exhibiting only 45% identity [26]. Zebrafish also possess three *CYP8Bs *(*CYP8B1*-*CYP8B3*), which are single-exon genes located on Chromosome 2 that share synteny with the single-exon human *CYP8B1*.

#### CYP11 (pregnenolone and aldosterone synthases)

Zebrafish have 2 *CYP11A *genes *(side-chain cleavage enzymes)*, *CYP11A1 *and *CYP11A2*, located adjacent to one another on chromosome 25, and one *CYP11B*-like gene, termed *CYP11C1 *(on Chromosome 16). Vertebrate CYP11A1 synthesizes pregnenolone from cholesterol, and a maternally-derived *CYP11A1 *mRNA has been suggested to form pregnenolone important in cell migration in the zebrafish zygote [27]. Following the onset of embryonic transcription, *CYP11A1 *is expressed in the yolk syncytial layer. It is then expressed in the embryonic interrenal primordia, and in the interrenal glands into adulthood [5,27,28]. In adult zebrafish it is expressed in the gonads, brain, and interrenal glands. Knockdown of zebrafish *CYP11A1 *leads to a shortened axis and epiboly defects (cell migration defects). Zebrafish CYP11A2 is 80% identical to CYP11A1 at the amino acid level, but little is known about functional similarities between the two forms, or about *CYP11A2 *expression, although based on our microarray results (see below) it appears to be expressed in the early embryo, and database EST evidence indicates that it is also expressed in adult gonads.

Zebrafish CYP11C1 exhibits 42% amino acid identity to human CYP11B2 (aldosterone synthase), but does not share synteny with any tetrapod CYP11B genes.

#### CYP17 (steroid 17α-hydroxylase/17,20-lyase)

CYP17 enzymes catalyze dual functions of steroid-17α-hydroxylase and steroid-17, 20-lyase. The zebrafish genome has two *CYP17A *genes that are 49% identical at the amino acid level, and the two genes are located on different chromosomes (Chr 13 and 23). One of these (*CYP17A1*) was cloned from zebrafish ovary [29]. *CYP17A1 *is expressed in gonadal tissue, but also in brain, gill, liver, and intestine. Zebrafish *CYP17A1 *is upregulated by fluorotelomer alcohols [30], but not by benzo[a]pyrene [31]. The functions of the two CYP17 s may differ (see Discussion, below) [32,33].

#### CYP19 (aromatase)

Zebrafish have two distinct *CYP19 *(aromatase) genes, *CYP19A1 *and *CYP19B1 *reported initially by Kishida and Callard [34]. The two aromatase forms are expressed in different tissues; *CYP19A1 *is expressed principally in the ovary, but also at much lower levels in the testis, while *CYP19B1 *is expressed in brain and neural tissue, including in the olfactory bulb, ventral telencephalon, preoptic area, and ventral/caudal hypothalamic zone, and in the anterior and posterior lobes of the pituitary [35]. Estrogen-mediated upregulation has been observed only for the neural form, *CYP19B1*, in adults and in embryos after 24 hpf [34,36].

#### CYP20A1 (undetermined function)

Zebrafish have one CYP20 gene, *CYP20A1*, 61% identical to the *CYP20A1 *in humans, and they share synteny (Table 3), both being adjacent to *ABI2*. At present, the substrate specificity and biological function of CYP20A1 are unknown, and it remains an "orphan" CYP [37]. However, there is evidence for high levels of mRNA expression in the hippocampus and substantia nigra of mouse [38], suggesting functions in these parts of the brain implicated in important neuropathies.

#### CYP21 (steroid 21-hydroxylase)

Zebrafish CYP21A1 shares 39% identity with human *CYP21A2*, and shares synteny with the human *CYP21A *gene pair (human *CYP21A1P *is a pseudogene). Mammalian CYP21A1 and CYP21A2 convert progesterone to deoxycorticosterone via 21-hydroxylation, prominently in the adrenal gland. The zebrafish CYP21A1 presumably serves a similar function, in the interrenal.

#### CYP24 (Vitamin D3 metabolism)

CYP24A1 is a mitochondrial enzyme catalyzing 1α, 25-dihydroxyvitamin-D3-24-hydroxylase. There is one *CYP24A1 *gene in zebrafish, with 63% percent amino acid identity to human CYP24A1. The zebrafish enzyme is presumed to function similarly to mammalian CYP24 s, as vitamin D3 and various metabolites circulate in fish serum [39] and zebrafish have a functional vitamin D receptor (VDR; NR1I1) with ligand specificity similar to other vertebrate VDRs [40].

#### CYP26 (retinoic acid hydroxylase)

There are three CYP26 s in zebrafish. *CYP26 *was first discovered in zebrafish [41] and the retinoic acid metabolizing function appears to be conserved throughout the vertebrates [42-44]. Each zebrafish *CYP26 *is located on a separate chromosome, suggesting early establishment of these duplicated genes. The *CYP26 s *are categorized into three separate subfamilies, with some confusion as to the proper classification, i.e., zebrafish *CYP26C1 *has previously been referred to as *CYP26D1 *[45]. *CYP26A1 *and *CYP26B1 *share synteny with human *CYP26A1 *and *26B1 *genes, respectively, while the *CYP26C1 *syntenic relationship is less distinct (Table 3). The three zebrafish *CYP26 s *are all induced by and metabolize retinoic acid, and play essential roles in hindbrain patterning [13,44-46]. Zebrafish CYP26 s also play roles in osteogenesis [2,47], in pancreatic development [48], and presumably other processes.

#### CYP27 (vitamin D3 metabolism)

Zebrafish have seven distinct *CYP27 *genes, distributed into three subfamilies, while in most mammals there are two or three *CYP27 *genes. Human CYP27A1 and CYP27B1 catalyze vitamin D3 25-hydroxylase and 25-hydroxyvitamin D3 1α-hydroxylase, respectively [22]. The five zebrafish *CYP27A *genes are all tandemly located on Chromosome 9 and share synteny with human *CYP27A1 *(Table 1). Zebrafish *CYP27B1 *and *CYP27C1 *are located on different chromosomes (Chr 11 and 6, respectively; Table 1). Functional properties of the various zebrafish CYP27 s are unknown.

#### CYP39 (24-OH-cholesterol-7α-hydroxylase)

A *CYP39A1 *is present in the zebrafish genome, located on chromosome 20. In mammals CYP39A1 functions as an oxysterol-7α-hydroxylase, involved in the conversion of cholesterol to bile acids. *CYP39A1 *has not been found in other published fish genomes, but is present in tetrapod genomes, with conserved synteny to human *CYP39A1*. Zebrafish *CYP39A1 *is approximately 4Mb from a cluster of genes that share synteny with human *CYP39A1*.

#### CYP46 (cholesterol 24-hydroxylase)

Zebrafish have four *CYP46A *genes. One (*CYP46A1*) is on Chromosome 20, two (*CYP46A2 *and *CYP46A4*) are arranged in tandem on Chromosome 5. A fourth (*CYP46A5*) is supported by EST data but missing from the current assembly (Zv8). In contrast, humans have one CYP46, CYP46A1. Human CYP46A1 functions both as a cholesterol 24(S)-hydroxylase and as a 24-hydroxycholesterol-hydroxylase [49,50]. CYP46A1 in mammals is a brain-specific cholesterol-metabolizing enzyme, important for maintaining brain cholesterol homeostasis and membrane function, but also with potential regulatory roles as a neurosteroid-metabolizing enzyme [51].

#### CYP51 (lanosterol 14α-demethylase)

Zebrafish possess one CYP51 gene, *CYP51A1*. In other animals CYP51 is a lanosterol-demethylase enzyme, the only CYP involved in the post-squalene portion of the cholesterol biosynthesis pathway. CYP51 is an essential enzyme in the *de novo *synthesis of cholesterol [52]). CYP51 is often considered to be a "housekeeping gene" based on its ubiquitous expression and conserved function [53]. Zebrafish *CYP51 *has been cloned and heterologously expressed in *E. coli *[54]. Microarray analysis and *in situ *hybridization of embryonic zebrafish showed that *CYP51 *is strongly expressed in the eye, in the brain, and in epidermal cells of the forehead and the tail fin [55], and suggesting a role for CYP51 and other isoprenoid synthesis enzymes in hematopoietic and vascular development.

### CYP Families 1-4 (Xenobiotic Metabolizing CYPs)

**Figure 2:**
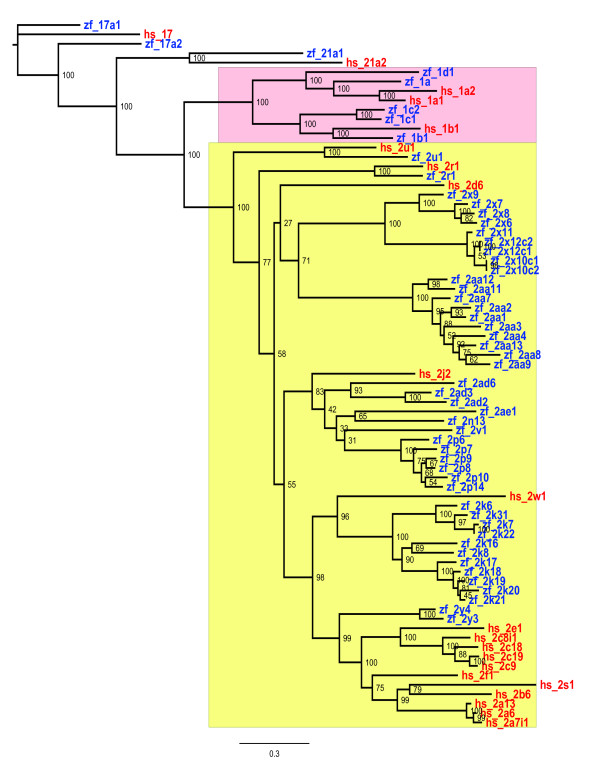
**Maximum likelihood phylogenetic tree of zebrafish and human Clan 2 amino acid sequences**. CYP1 family sequences are highlighted in red, while CYP2 sequences are highlighted in yellow. CYP2R1 and CYP2U1 are conserved. Zebrafish CYP2J co-orthologs clearly cluster with the human CYP2J, as do the zebrafish CYP2Ks with human CYP2W1, and zebrafish CYP2Ys with most of the remaining human CYP2 s. CYP1 evolution has been described elsewhere [58,59]. Human (hs) sequences are red, zebrafish (zf) are blue.

CYP families 1, 2, 3, and 4 include enzymes involved in drug, xenobiotic and fatty acid metabolism. In contrast to the CYPs that have principally endogenous functions, these CYP genes are much more diverse, with much less conservation of sequence between zebrafish and human. Table 2 provides specific information on the CYP 1-4 family genes. A molecular phylogeny of Clan 2 genes, which includes families 1 and 2 as well as CYP17 and CYP21, is shown in Figure 2. Molecular phylogeny of the Clan 3 genes, including the CYP3 s and CYP5 s (discussed above) and Clan 4 genes (the CYP4s) can be seen in Figure 1. Some relationships between zebrafish and human CYPs in families 1-3 suggested by molecular phylogeny have been substantiated by synteny analysis.

#### Clan 2- CYP1s

Zebrafish have five *CYP1 *genes in four subfamilies, *CYP1A*, *CYP1B1*, *CYP1C1*, *CYP1C2*, and *CYP1D1*, all of which have been cloned and sequenced [56-59]. Zebrafish *CYP1A *has exon structures similar to the human *CYP1A1 *and *CYP1A2*, and the single *CYP1B1 *gene has a gene structure very similar to human *CYP1B1*. The *CYP1Cs *are closely linked single-exon genes [57,60]. The recently identified zebrafish *CYP1D1 *has a gene structure identical to zebrafish *CYP1A*, and quite different from the *CYP1B1 *and *CYP1Cs *[59]. Members of all four CYP1 subfamilies have been identified in other fishes and non-mammalian tetrapods, and the evolution of the *CYP1 *family has been discussed elsewhere in some detail [59,60]. Notably, the *CYP1Cs *are absent and *CYP1D1 *is a pseudogene in human and some other mammals [59,61]. *CYP1A*, *CYP1B1 *and the *CYP1Cs *are inducible by AHR agonists [58,62]. Vertebrate CYP1As and CYP1B1 generally are involved in the metabolism of various drugs, many hydrocarbons, steroids and fatty acids. With the exception of benzo[a]pyrene and estradiol [63,64], little is known of the possible xenobiotic and endogenous substrates of the CYP1Cs and of CYP1D1.

#### Clan 2 - CYP2s

As in mammals, the CYP2 s constitute the largest CYP gene family in zebrafish, with 47 CYP2 genes, in contrast to 16 in humans. The subfamilies are considered below, in order according to suggested relationships: (i) orthology indicated by sequence, (ii) homologs suggested by sequence and orthology confirmed by shared synteny, and (iii) no evident homologous relationship to human CYPs.

##### (i) CYP2R and CYP2U

There are single genes in each of these two subfamilies in zebrafish, *CYP2R1 *and *CYP2U1*. These can be classified as orthologs of human *CYP2R1 *and *CYP2U1*, based on sequence identity. They also exhibit shared synteny with their respective human counterparts (Table 3). CYP2R1 is a microsomal vitamin D 25-hydroxylase in humans [65,66]. Human CYP2U1 is expressed in the brain and the thymus [67], and catalyzes ω and ω-1 hydroxylation of fatty acids, including arachidonic acid [68]. CYP2U1 appears to have a pre-vertebrate origin, and may be the oldest identifiable vertebrate CYP subfamily [69].

##### (ii) CYP2N, CYP2P, CYP2V, CYP2AD

**Figure 3:**
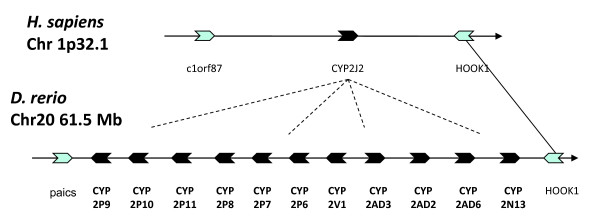
**Syntenic arrangement of the human and zebrafish CYP2J locus**. Human CYP2J2 metabolizes arachidonic acid to biologically active eicosanoids, some of which have vasoprotective effects. All vertebrate genomes that have been examined have at least one gene at this locus; some have many tandemly duplicated CYPs. Fish CYP2J orthologs are not members of the same subfamily, and functions may have diverged somewhat. Gene order was obtained from Ensembl 58.

The 11 zebrafish genes in these five subfamilies occur in a clade together with human *CYP2J2 *(Figure 2). Analysis of gene location showed that the six *CYP2Ps *and *CYP2N1*3, *CYP2V1*, *CYP2AD2*, *CYP2AD3*, and *CYP2AD6 *all are arranged in tandem on chromosome 20. This cluster of CYP genes shares synteny with human *CYP2J2*, indicating that all 11 zebrafish genes at this locus share an ancestral origin with *CYP2J2 *(Figure 3). CYP2J2 is an arachidonic acid epoxygenase with roles in the cardiovascular system. Functions of the members of the zebrafish cluster are unknown, although related genes in killifish (CYP2P3 and CYP2N1 and 2N2) are arachidonic acid hydroxylases and CYP2P3 in particular is similar in function to human CYP2J2 [70,71].

##### CYP2K

Eight *CYP2K *genes are present in a tandemly duplicated array on Chromosome 3. This cluster of *CYP2Ks *shares synteny with human *CYP2W1 *(Additional File 1, Figure S2). Human CYP2W1 is a tumor-specific CYP [72,73] that oxidizes indole and chlorzoxazone, but not fatty acids [74]. *CYP2K6*, which is not highly expressed until 5 dpf, has been heterologously expressed, and demonstrated to catalyze the activation of the mycotoxin aflatoxin B1 (AFB1) to the carcinogenic *exo*-8,9-AFB1 epoxide [75]. The low level *CYP2K6 *expression during zebrafish embryo and larval stages may help to explain the lack of AFB1 toxicity in zebrafish embryos [75]. Functions of the other CYP2Ks are not known.

##### CYP2Y

*CYP2Y3 *and *CYP2Y4 *are tandemly arranged on chromosome 15. They share synteny with a cluster of *CYP2 *genes for human drug-metabolizing enzymes, including *CYP2A6*, *CYP2A13*, *CYP2B6*, *CYP2F1*, and *CYP2S1 *located on human chromosome 19 (Additional File 1, Figure S3). The human genes at this locus include genes induced by PXR agonists (CYP2B6) and genes induced by AHR agonists (CYP2S1). Whether CYP2Y3 and CYP2Y4 have functional or regulatory properties in common with any of these human CYPs is not known.

##### (iii) CYP2X

There are nine *CYP2X *genes present in two separate arrays of tandemly duplicated genes, on Chromosomes 7 and 25. Two of the *CYP2X *genes (*CYP2X10 *and *CYP2X12*) (Table 2), have two exact copies in the current genome assembly (Zv8; Ensembl Build 58) likely as an artifact of the assembly process, so there is some uncertainty as to the exact number of CYP2Xs. The *CYP2X *genes do not appear to share synteny with any mammalian CYP. Catalytic activities of CYP2Xs in zebrafish are unknown, although *CYP2X1 *from channel catfish has been cloned [76] and heterologously expressed [77]. Aminopyrine and benzphetamine demethylase activities were observed, but no biological function has as yet been ascribed.

##### CYP2AA

Due to overlapping gene predictions, the current genome assemblies and gene counts are not accurate in the region on chromosome 23 where the *CYP2AA *genes are located. Ten *CYP2AA *genes are present in a tandemly duplicated array on chromosome 23. GeneWise [78] predictions indicate that there are 10 *CYP2AAs*, with high identity to each other (65-85%), but no clear orthology to other CYP2 s. At least three have been cloned (*CYP2AA1*-*2AA3*; Bainy et al., Buhler et al., unpublished data). Some *CYP2AA *genes appear to be induced by PXR agonists (Kubota et al., unpublished data). The CYP2 s most closely related to the *CYP2AAs *are the *CYP2Xs*, and *CYP2R1 *(Figure 2).

##### CYP2AE

The two *CYP2AE *genes are present as tandem duplicates on Chromosome 23, approximately 230 kb downstream from the *CYP2AA *cluster. No shared synteny is evident with mammalian genomes, and is unclear even for other published fish genomes. However, the *CYP2AEs *cluster phylogenetically with the *CYP2J *orthologs *CYP2N/P/V/AE *(Figure 2), suggesting a possible insertion of a set of duplicated *CYP2J *orthologs.

#### Clan 3 - CYP3s

The zebrafish genome includes five *CYP3 *genes, *CYP3A65*, and four *CYP3C*s, *CYP3C1*-*3C4 *(Table 2).*CYP3A65 *and *CYP3C1 *were previously cloned and heterologously expressed [79,80]. *CYP3A65 *is a13 exon gene located in chromosome 1, and is 54% identical to human CYP3A4. Zebrafish *CYP3C1*-*CYP3C4 *are all 13-exon genes located in tandem on zebrafish chromosome 3 (Table 1). Other fish species also have multiple *CYP3 *genes located in this region (e.g. four in stickleback and one in fugu), and chickens appear to have two homologs at that locus. It is likely that the previously named human pseudogenes *CYP3A*-*se1 *and *CYP3A*-*se2 *[21] are the orthologous remnants of these *CYP3s*, as they are located in the same region (on chromosome 7q22.1) as the immediately adjacent *FOXK1*gene (Additional File 1, Figure S3). Qiu et al. (2008) found that in most vertebrates apparently intact (i.e. not obviously pseudogenized) CYP3 genes are located within one of two genomic regions, labeled *CYP3HR1 *and *CYP3HR2 *[81]. Zebrafish *CYP3Cs *are within *CYP3HR1*, while the human *CYP3As *are in *CYP3HR2*. Zebrafish *CYP3A65 *does not share synteny with CYP3 s in other fish for which genomic information is available (medaka, stickleback, fugu, or tetraodon). *CYP3A65 *is inducible by some PXR agonists [80,82]. Other fish CYP3As are prominent in catalyzing testosterone 6β-hydroxylase [83], and this is likely true for zebrafish CYP3A65 as well. Activities are not known for the other zebrafish CYP3 s.

#### Clan 4 - CYP4s

There are four CYP4 genes in zebrafish, *CYP4F43*, *CYP4T8*, *CYP4V7*, and *CYP4V8 *(Table 2), in contrast to humans, which have 12 CYP4 s. Mammalian CYP4F enzymes function as omega-hydroxylases of C_16_-C_26 _fatty acids, including eicosanoids such as leukotriene B4 [84,85]. The full range of substrates of CYP4V enzymes is not yet known, although human CYP4V2 is a selective omega-hydroxylase of saturated, medium-chain fatty acids [86]. The CYP4 s appear to have less involvement in xenobiotic metabolism than many CYP1 s, CYP2 s or CYP3 s, although some xenobiotics (e.g., phthalates and perfluorooctanoic acid) induce *CYP4Ts *possibly via peroxisome proliferator-activated receptor alpha (PPARα) and gamma (PPARγ) [87,88]. Zebrafish *CYP4F43 *and the *CYP4Vs *share synteny with their human counterparts, but zebrafish *CYP4T8 *does not occupy a shared syntenic position with other CYP4 s.

### Developmental Expression

**Figure 4:**
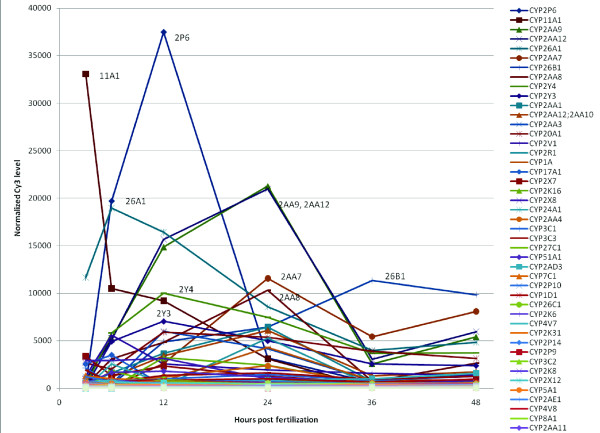
**Expression of 88 CYP genes in zebrafish during the first 48 hours of development**. Single color microarray analyses of CYP gene expression throughout early development (3-48 hours post fertilization, hpf) shows that while some CYP genes are expressed in the whole embryo at high levels, most CYP genes are expressed at levels significantly above background (~5 fluorescent units; see Additional File 2, Table S3). Strong temporal signals are apparent.

**Figure 5:**
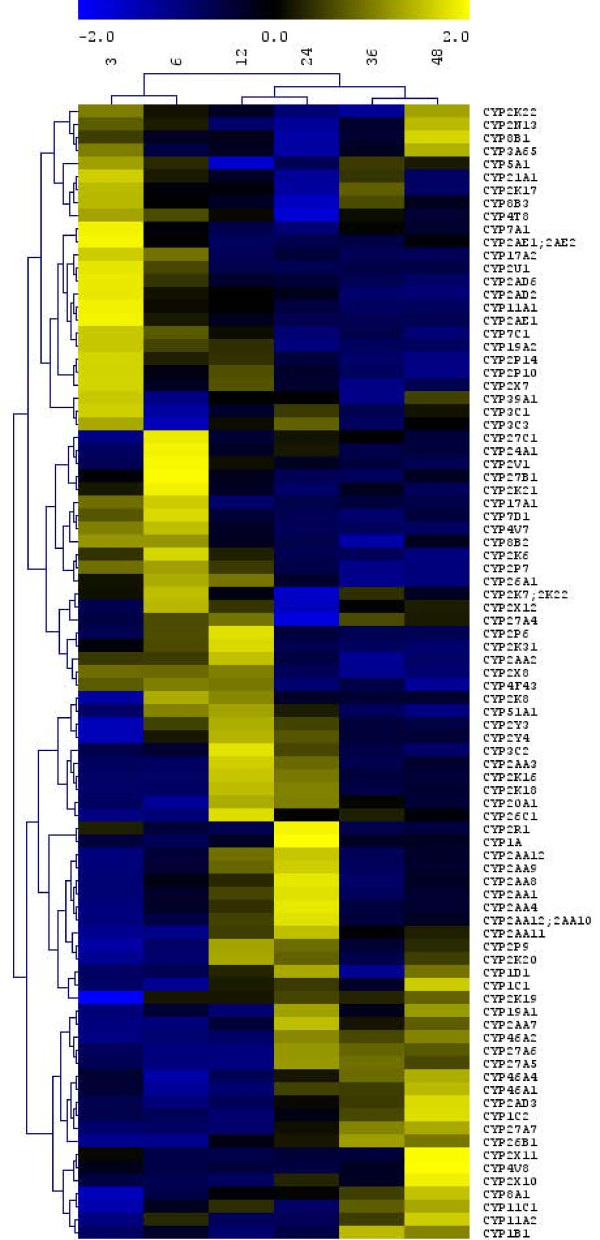
**Heat map of CYP developmental expression with each gene z-normalized to display the temporal expression waves**. Hierarchical K-means clustering of z-normalized data exposes the temporal waves of CYP gene expression through early zebrafish development. Different clusters of genes are expressed at the different sampling times. A few sets of genes exhibit bimodal distributions, particularly CYP3A65, 8B1, 2N13, 2K22, and to a lesser extent, CYP21A1, 2K17, 8B3, 27A4, and 5A1. Unnormalized clustering presents a different pattern, as high-expressing genes tend to be clustered together (Additional File 1, Figure S6). CYP2AA6, CYP2AA13, CYP2P8, CYP2X6, CYP2X9, and CYP46A5 are not represented on the microarray, as the microarray was generated using Zv6 rather than the current genome assembly.

Using single-color Agilent custom whole-transcriptome microarrays, we analyzed the expression of 88 CYP genes over the course of early development in zebrafish (plot, Figure 4; heat map of normalized values, Figure 5). Zebrafish embryos were staged and sampled from 3 hours post-fertilization (hpf) through 48 hpf, and four biological replicates were sampled and analyzed (see Methods). After normalization, probes exhibiting saturation or signals that were not above background were removed (10 and 82 probes, respectively), leaving 21801 probes for examination of expression patterns. None of the excluded probes were for CYPs, indicating that all 88 *CYPs *examined were expressed during development. Bayesian Estimation of Temporal Regulation (BETR; [89]) showed 15393 significant differentially expressed probes (p < 0.01), similar to the results of a naïve ANOVA analysis. Of the 88 CYP genes examined, 22 were found not to have differential expression during development. All others had one or more probes that indicated significant differential expression between sampling times.

**Figure 6:**
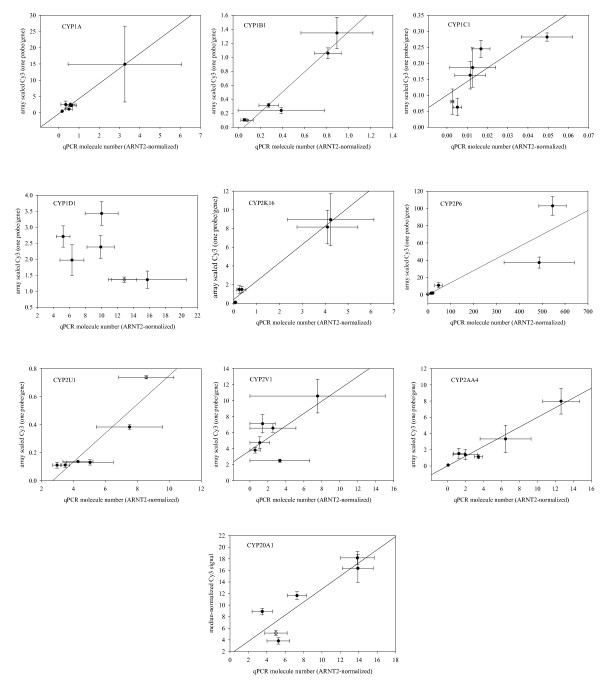
**RT-qPCR analysis of CYP expression for 12 genes**. CYP1A, 1B, 1C1, 1D1, 2K16, 2P6, 2U1, 2V1, 2AA4, 2AA6, 2AA11 and CYP20A1 were examined. qPCR confirms the microarray results for 10 of the 12 CYP genes examined. Both microarray and qPCR analyses were normalized by ARNT2 expression. Only CYP1D1 and CYP2AA4 exhibit deviations from linearity.

Expression patterns of a subset of 11 *CYPs *were assessed using qPCR. Molecule counts were calculated by using plasmid-derived standard curves, and expression levels were normalized to *ARNT2*. We previously observed [62] that *ARNT2 *has significantly less variability during development than β-actin, a conclusion supported here by the single-color microarray data (Additional File 1, Figure S4). Nine of the eleven CYPs examined with qPCR exhibited linear relationships between the *ARNT2*-normalized microarray data and the *ARNT2*-normalized qPCR molecule counts (Figure 6).

### Patterns of Expression

Several zebrafish CYPs exhibited expression levels that varied by 10 fold or greater (from low to high point) during development. Among these are *CYP2P6*, *CYP11A2*, *CYP26A1*, *CYP26B1*, *CYP2AA7-CYP2AA9, CYP2AA12*, *CYP2Y3*, *CYP2Y4*, and *CYP20 *(Figure 4). Some had highest levels of expression at the earliest time point (3 hpf). Others showed a peak at intermediate times, and still others showed elevated expression at the latest time sampled (48 hpf). Previous investigators have observed similar variation in expression levels for some of these CYPs, including *CYP2P6 *[90], *CYP11A1 *[28], and the *CYP26 s *[13,45].

An alternate way of depicting developmental expression is a within-gene normalized heat-map, which shows the relative expression of all CYPs over the course of development (Figure 5). While the absolute expression levels of different genes may be low, especially in comparison to "housekeeping" genes such as *β-actin *or *ARNT2*, within-gene normalization can reveal the relative changes in gene expression throughout the time series, and allow more ready comparison of profile clusters. This approach revealed a striking picture of waves of CYP gene expression as development progresses. The waves of CYP expression occurred with different clusters expressed most strongly usually only at one or two sequential time points (i.e., unimodally) during development. *CYP2K22*, *CYP2N13*, *CYP3A65*, and *CYP8B1 *appear to have bimodal expression, with elevated levels both at 3 and 48 hpf (Figure 5). A similar bimodal expression pattern was seen with *CYP5A1*, *CYP21A1*, *CYHP2K17*, *CYP8B3*, and *CYP4T8*, which had the highest relative expression at 3hpf and 36 hpf.

Clustering of gene expression patterns (K-means clustering) revealed co-expression of tandemly duplicated CYPs, evident in the expression clustering of subsets of the *CYP2AAs*, the *CYP2Ks*, the *CYP2Ys*, the *CYP46As*, and the *CYP27As *(Figure 5). However, other clusters, such as the set of CYP2 subfamilies that share synteny with human CYP2J2 did not exhibit complete co-expression: CYP2P10, CYP2P14, CYP2AD2, and CYP2AD6 were mostly highly expressed at 3 hpf, while CYP2P6, CYP2P7, and CYP2V1 were most highly expressed at 6-12 hpf.

**Figure 7:**
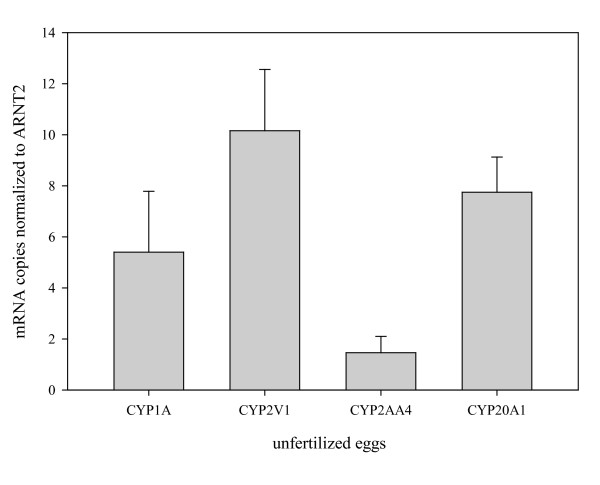
**Expression of four CYP genes in unfertilized oocytes**. The maternal contribution to transcript abundance of selected CYPs was determined using qPCR on unfertilized oocytes. Eggs were expressed from gravid female zebrafish (n = 3) by gentle squeezing of anesthetized fish. Data was normalized using ARNT2.

The observation that some *CYPs *showed high levels of transcript at 3 hpf, implies that these could involve maternally-derived transcript in oocytes. A number of CYP genes identified by cluster affinity search analysis using MeV [89,91] appear to have a maternal signal in the microarray data, based on the decreasing expression levels from 3 hpf (Additional File 1, Figure S5 and Additional File 2, Table S1). To begin assessing maternal transcript we examined selected CYP in unfertilized eggs. qPCR analysis of unfertilized eggs showed that *CYP1A*, CYP2V1, *CYP2AA4*, and *CYP20 *had significant *ARNT2*-normalized expression levels in unfertilized eggs (Figure 7). Analysis of the larger complement of CYP genes for maternal transcript in unfertilized eggs requires microarray analysis, and is ongoing.

## Discussion

In this study we identified the total CYP complement in zebrafish, and assessed patterns of expression of these genes during normal embryonic development. Zebrafish are an increasingly important model in developmental toxicology, pharmacology and chemical effects on disease. Knowing the identity and regulation of CYP genes is essential to strong inference regarding chemical effects in this model, and to assess pathways of metabolism of xenobiotics and endobiotics, and the relationship to CYP roles in these processes in humans. The 94 zebrafish CYPs occur in the same 18 gene families that are found in humans and other mammals, but with differences in numbers of genes, and often with uncertain function.

### CYP Families 5-51

Many of the CYP gene families in this group have single genes in zebrafish, as they do in humans, and show a high degree of conservation of sequence with their human counterparts. These genes also exhibit similar syntenic relationships as found in human. Together, the sequence data and gene location data indicate that in many of these families the genes are direct orthologs of their human (mammalian) counterparts. Where there is 1:1 correspondence in these gene families, i.e., with CYP5A1, 7A1, 7B1, 8A1, 20A1, 21A1, 24A1, 26A1, 26B1, 39A1, 46A1 and 51A1, it most likely indicates conservation of enzyme activities and physiological function. In other gene families in the "endogenous set", defining relationships between human CYPs with important endogenous functions and the zebrafish homologs is complicated by the presence of multiple closely related paralogs in zebrafish, not found in human. Thus, zebrafish have two CYP17As, two CYP19As, and four CYP46As, while humans have only half that number in each case. Such doubling of the numbers of genes in several CYP gene families could be the result of individual gene duplication, or could be remnants of the third round of whole genome duplication (WGD-3 [24,92]), with the retention of duplicated genes in zebrafish.

Zebrafish CYP paralogs that are co-orthologs of the human CYPs could have distinct functions, as a result of function partitioning (subfunctionalization) or differential regulation (temporal and/or organ differences or differences in induction). This has been observed for *CYP19*, with *CYP19A1 *(ovarian) and *CYP19A2 *(brain) aromatases displaying distinct expression patterns and inducibility. The neural form (*CYP19A2*) exhibits sensitivity to induction by estrogen, while the ovarian form appears to be mostly recalcitrant to induction by estrogen receptor agonists [36,93-95]. While functions have not been confirmed for zebrafish, the two *CYP17A *genes in other fish also appear to represent enzyme sub-functionalization following gene duplication. Tilapia and medaka CYP17A1 possess both steroid-17α-hydroxylase and 17, 20-lyase activities, as does mammalian CYP17A1, but both tilapia and medaka CYP17A2 possess only 17α-hydroxylase activity, as they only convert pregnenolone or progesterone to 17α- hydroxy products, but do not perform the subsequent conversion to androstenedione or DHEA [32,33]. There also are significant differences in spatial expression patterns for the duplicated *CYP17A *genes during development, and during the spawning cycle in other fish [32]. We did not see any substantial temporal separation of *CYP17A1 *and *CYP17A2 *expression during development, although *CYP17A1 *is more strongly expressed (Figure 4 and Additional File 2, Table S2).

In some families of "endogenous" genes, zebrafish have more than twice the number of genes than occur in humans, and some have novel subfamilies as well. This is evident in the *CYP27s*, and the *CYP46s*, where the numbers of genes are greater than would be expected to have resulted from WGD-3. It is likely that the ancestral condition would be one of fewer genes, with expansion in zebrafish rather than loss in humans. That expansion in zebrafish could involve WGD, but is most evident in tandem duplication as well as translocation. This is clearly suggested in CYP27 by the four genes that share synteny with the single human *CYP27A1*, and the presence of *CYP27B1 *and *27C1*, which do not share synteny with human CYPs. The biological significance of some duplicated genes also could involve distinctions in temporal or organ- and cell-specific regulation, but determining this can be complicated by the strong possibility of substrate overlap.

### CYP Families 1-4

Zebrafish CYP genes in families 1, 2, and 3 are more diverse than in humans, and sequence identities often are too low to discern orthology between zebrafish and mammalian genes in these families. However, analysis of the additional character of shared synteny clarifies evolutionary relationships between human and zebrafish genes in these families. CYP family 4 differs from families 1-3, as there are fewer CYP4 genes in zebrafish than there are in mammals. However, like the CYP1 s, CYP2 s and CYP3 s, the CYP4 s also are involved with (induced by or metabolize) xenobiotics, while this is seldom the case with CYP5-CYP51 genes.

Consistent with phylogeny, the fish CYP1 and CYP3 clades appear as sister groups to the mammalian clades for these genes. However, as discussed earlier [60], both mammals and fish share the CYP1 subfamilies CYP1A and CYP1B. Zebrafish also express CYP1Cs, which do not occur in humans, and CYP1D1, a pseudogene in human. All mammalian CYP3 s are in a single subfamily, CYP3A, which occurs in zebrafish as well. However, zebrafish CYP3A65 shares synteny with single exon pseudogene CYP3As in human (*CYP3A-se1 *and *-se2*), while the novel CYP3C subfamily [79] shares synteny with the functional human CYP3A3, 3A4 and 3A7 [81]. Fugu and possibly other fishes also have a second CYP3 subfamily, CYP3B, about which nothing is known [96,97].

The functional similarities in different taxa, suggest similar biological roles for the homologous CYP1 s and CYP3 s. Thus, CYP3As are the primary catalysts of testosterone 6β-hydroxylase in fish and mammals [98], and mammalian and fish CYP1As and likely CYP1Bs are prominent in the metabolism of some PAH pro-carcinogens. The roles of orthologous CYPs in metabolism of particular compounds can differ between taxa, however. Thus, the regio-specific oxidation of BaP and the rates of metabolism of planar HAH appear to differ in degree between fish and mammalian CYP1As [99,100], apparently reflecting species differences in CYP1A structures [101]. The functions of the novel CYP1 s and CYP3 s are less well defined, although zebrafish CYP1Cs and CYP1D1 have been expressed and functions have been determined with BaP [64], estradiol [63], and a number of other exogenous and endogenous substrates (Urban, Stegeman, et al. unpublished results). Little is known of the function of the CYP3Cs, but *CYP3C1 *appears not to be responsive to chemicals that induce *CYP3A65 *[79].

Identifying zebrafish-human orthologs is most difficult in the CYP2 family, where the differences in CYP2 divergence between zebrafish and humans obscure many homologous relationships. Thus as noted, of the 11 zebrafish and 11 human CYP2 subfamilies, only two (CYP2R and CYP2U) warrant the same designation in zebrafish as in humans based on sequence identity. While the disparity between zebrafish, or other fish, and mammals is exaggerated by evolutionary distance, our analysis of shared synteny indicates that members of distinct subfamilies in mammals and fish still may bear co-orthology. The CYP2J-related genes are a key example. The single clade of 11 genes in the zebrafish *CYP2N*, *2P*, *2V*, and *2AD *subfamilies and human *CYP2J2 *(Figure 2), implies relationship between the fish and human genes. Our analysis shows the zebrafish genes occur in tandem in a cluster that shares synteny with *CYP2J2*, indicating co-orthology (Figure 3). This suggests that there are catalytic functions among these zebrafish CYPs that are similar to the human CYP2J2. A similar hypothesis was borne out in functional characterization of previously identified killifish CYP2P3, which also clusters with the CYP2Js on phylogenetic analysis. Heterologously expressed CYP2P3 exhibited nearly identical regio- and stereoselectivity for oxidation of arachidonic acid as human CYP2J2, consistent with molecular phylogeny indicating a shared ancestral origin [71]. Notably, at present there is little or nothing known about the catalytic or biological functions or the chemical regulation of the majority of zebrafish CYP2 s.

### Zebrafish CYPs in development

In addition to annotating the full complement of CYP genes, we analyzed the expression of 88 CYP genes over the course of development in zebrafish (Figure 4). Several of these zebrafish CYPs exhibited markedly elevated expression levels at some time during development, but a large number, including more than three-quarters of the total genomic complement, showed distinct temporal expression patterns (Figure 5, Additional File 1, Figure S6). Importantly, gene expression profiling performed on whole embryos often under-estimates the importance of tissue- or cell-specific gene expression due to dilution effects.

Our array results are similar to developmental expression that has been determined for some individual CYP genes (e.g. CYP1 s [59,62], CYP2K6 [75], CYP3C1 [79], CYP19 [36,102]). Developmental roles have been established principally for some of the "endogenous" CYPs. Such genes include CYP11A1, which is essential for the synthesis of pregnenolone, critical for cell migration [27]; and the three CYP26 s, which contribute to retinoic acid gradients that regulate hindbrain and neural crest patterning [13,103,104] and osteogenesis [2,47]. We and others have seen complex CYP expression patterns in development. Developmental roles of CYPs in families 1-3 are unknown, although morpholino knockdown of CYP1Cs appears to protect from developmental toxicity of dioxin (Kubota et al., unpublished data) and over-expression of CYP2P6 caused developmental abnormalities, including cardiovascular abnormalities [90], suggesting developmental significance of these genes.

The roles for many CYPs, including roles in development, cannot necessarily be inferred from sequence identities. Thus, CYP20A1 is an 'orphan' CYP that does not have a defined function in zebrafish or in humans [37,38], and some large subfamilies (CYP2X, CYP2AA) do not have any homologs in mammals. As well, the different numbers of genes that are co-orthologs of single human CYPs precludes assignment of a function to any one, which requires empirical determination. This is true for the multiple co-orthologs in the "endogenous" CYP families, such as the CYP11 s, the CYP27 s and CYP46's, as well as for most those in the "xenobiotic" CYP families. The issues in CYP11 exemplify the questions and approaches. Zebrafish *CYP11A1 *is expressed throughout development, as in the murine model, but the *CYP11A1 *knockdown is not lethal [5,27]. As zebrafish have two *CYP11A *genes and a *CYP11C *gene, it is possible that overlapping substrate specificity and spatiotemporal expression patterns might allow one to substitute for the other in loss-of-function studies.

There is a greater dearth of information regarding CYP genes that may have maternally derived transcripts deposited in oocytes. Our analysis of transcripts of a few selected CYP genes, CYP1A, CYP2V1, CYP2AA4, and CYP20A1, showed that transcripts for all four were present in unfertilized zebrafish eggs, and that the levels of transcript could be substantial. CYP19 mRNA also has been reported in oocytes [105], and *CYP1A *transcript also was reportedly recently by others [106]. The significance of maternal transcript of these CYPs is not known. Whether other CYPs also have maternal transcripts deposited in oocytes, and what influences that deposition, is under investigation.

## Conclusions

The identification of the full complement of 94 zebrafish CYPs, and determination that the majority of CYPs have distinct developmental patterns of expression opens the door to assessing their role(s) not only in development, but also in the response of embryos to toxic chemicals. For many CYP genes involved in xenobiotic metabolism, inferring relationships among the genes between zebrafish and human based on sequence is difficult, complicated by remnants of whole genome duplication in the teleost line, uneven gene duplication and gene loss in different animal and gene lineages, and the possibility of gene conversion of closely linked paralogs [107]. Nevertheless, shared synteny indicates orthologous relationships of many zebrafish and human CYPs, and also indicates which zebrafish CYPs have no apparent homologs in humans. The lineage-specific diversification makes possible the acquisition of new functions (sub-functionalization) and regulation. In most cases, however, functions of the individual paralogous CYPs in zebrafish have yet to be determined. Those functions, and the timing, location and magnitude of expression in development will determine the strength of inference from developmental toxicological studies with the zebrafish model.

## Methods

### Gene identification and sequence analysis

Cytochrome P450 genes were identified by hidden Markov model searches (HMMER v2.3.2; [108]) of the ENSEMBL Zv6 and Zv7 gene predictions, using the global PFAM model for cytochrome P450 s (p450-ls). Annotations were transferred to the Zv8 alignment when it became available. Alignments were constructed using Muscle v3.6b [109], and masked based on the Muscle alignment scoring function. Phylogenetic trees were constructed by analyzing inferred or confirmed amino acid sequences using maximum likelihood (RAxML 7.0.4, [110]). The WAG model of amino acid substitution [111] with a gamma distribution of substitution rates was used in all Bayesian analyses (WAG+G). Synteny was examined using the ENSEMBL and UCSC browsers.

### Animals

Tubingen long fin (TL) zebrafish were progeny of TLs obtained from the laboratory of Dr. Mark Fishman, crossed with TLs raised from eggs obtained from the Zebrafish International Resource Center at the University of Oregon (Eugene, OR, USA). Fish were maintained in the Woods Hole Oceanographic Institution Zebrafish Facility and the experimental procedures were approved by the Institutional Animal Care and Use Committee. The zebrafish were held in 2:1 female to male groups at a density of ≤5 fish/l in aerated, filtered and re-circulated system water (28.5°C) in 3 or 10 l tanks in an Aquatic Habitat™system. The system water was composed of Instant Ocean™(60 mg/l), sodium bicarbonate (50 mg/l), calcium sulfate (8.5 mg/l) and Kent's Freshwater Essentials™(53 μl/l) in distilled water. Fish were fed twice daily with brine shrimp (*Artemia salina*) and once daily with Omega One flakes (Omega Sea Ltd. Sitka, AK, USA).

### Zebrafish embryos

TL embryos were obtained from group breedings of 30 female and 15 male fish. Fertilized TL zebrafish eggs (100) were placed in 20 cm glass Petri dishes containing 50 ml of 0.3x Danieau's solution. The embryos were incubated at 28.5°C. At 24 hpf, the solutions were replaced with fresh 0.3x Danieau's solution and any dead embryos were removed; mortality was normally four or less per dish. For sampling, all embryos in a dish were pooled, frozen in liquid nitrogen, and stored at -80°C. Sampling times were 3, 6, 12, 24, 36, and 48 hpf. Each of four dishes was maintained independently, as biological replicates.

A separate collection of unfertilized eggs was taken to determine the maternal contribution to transcript abundance of selected CYP genes, which could imply function in early development. Eggs were expressed from gravid female zebrafish (n = 3) by gentle squeezing of anesthetized fish, following published protocols [112]. Eggs that showed development of a perivitelline space were considered mature eggs and were selected for analysis.

### Microarray

The Agilent (Agilent Technologies, Santa Clara, CA) 4 × 44k DNA gene expression microarray was used to probe developmental gene expression. The original Agilent gene set was missing many CYPs and other genes involved in chemical defense. Probes for these genes were custom designed using the Agilent eArray system and added to the array. The individual microarrays had 21893 unique probes (excluding controls) printed in duplicate.

Microarrays were used to examine gene expression levels for all four replicates of each timepoint (3, 6, 12, 24, 36, and 48 hpf). Total RNA was extracted using the Aurum Fatty and Fibrous Tissue kit (Bio-Rad, Hercules, CA). RNA samples were checked for quality using a NanoDrop ND-1000 spectrophotometer and an Agilent 2100 BioAnalyzer. For each RNA sample, a single microarray was hybridized with 750 ng Cy3 labeled cDNA using Agilent's standard conditions for single-color microarrays at the Whitehead Center for Microarray Technology. The Agilent Low-Input QuickAmp Labeling Kit was used for labeling, the samples were hybridized to a the custom Agilent 4 × 44K feature zebrafish microarray using the Agilent In situ Hybridization Kit Plus, and labeled cDNA was combined with the Agilent 10× Control Targets (to identify microarray corners). Post-hybridization, microarray slides were washed as per the Agilent In situ Hybridization Kit Plus. Arrays were scanned with an Agilent DNA Microarray Scanner.

### Microarray analysis

Analysis of raw microarray results was performed using Agilent's Feature Extraction software with background detrending (spatial and multiplicative). The data were then normalized using the non-linear scaling method based on rank invariant probes of Schadt et al. [113,114], commonly performed for Affymetrix microarray data by the dChip software [115] and performed for our Agilent data by software developed by AGM. Briefly, this method finds the microarray with the overall median Cy3 signal for use as a baseline microarray (here 12 hpf, replicate C) to which all other microarrays are normalized. For normalization, microarrays are each separately normalized to the baseline microarray by finding Rank Invariant probes between the two microarrays using the method outlined in Schadt et al. [113]. The software develops a non-linear normalization curve for each microarray and uses a piecewise linear running median to normalize the data. Prior to normalization any Cy3 values below 5 were set to 5 and after normalization the average normalized signal was calculated for probes duplicated on the microarray. Probes were removed from consideration when they exhibited saturation in any instance among the microarrays and duplicate probes, or when signals were not above background, in all instances among the microarrays and duplicate probes. Bayesian Estimation of Temporal Regulation (BETR; [89]) was used to analyze the developmental time series, as standard ANOVA is not appropriate due to auto-correlation between time points. Normalized Cy3 values for each probe were log transformed, median-centered, and analyzed using BETR relative to 3 hpf.

Gene expression clustering was analyzed using MeV (v4.3) [116]. Hierarchical K-means clustering of both un-normalized and within-gene z-normalized mean gene expression values (n = 4 biological replicates) was performed, assuming 10 clusters. A different measure of clusters was performed using the Cluster Affinity Search Technique [91], setting the cluster affinity to 0.7 (0-1 scale).

Microarray data has been deposited in the Gene Expression Omnibus (GEO) database (GEO accession number GSE24840).

### Real-time, quantitative RT-PCR

RNA quantity and quality were determined spectrophotometrically (NanoDrop ND-1000; NanoDrop Technologies, Wilmington, DE, USA). cDNA was synthesized using the Omniscript™ Reverse Transcriptase kit (Qiagen Inc., Valencia, CA, USA), random hexamer primers (Operon Biotechnologies Inc.) and the RNasin^® ^RNase inhibitor (Promega). Gene-specific PCR primers for zebrafish *CYP*, and *ARNT2 *were synthesized by Operon Biotechnologies Inc (Table S1). (The *ARNT2 *primers were designed to amplify a sequence common to *ARNT2a, b*, and *c *[58]). Real time PCR was performed using the iQ SYBR Green Supermix (Bio-Rad) as previously described [58,62]. To ensure that a single product was amplified, melt curve analysis was performed on the PCR products, and polyacrylamide gel electrophoresis was performed after the PCR run for each gene. The specificity of the CYP qPCR primer pairs was tested by purifying and sequencing the cDNA products of the final qPCR reactions.

A standard curve for each gene was generated by serially diluting plasmids containing the 100-150 bp region amplified by the quantitative PCR primers. Total molecule numbers were calculated for each sample and normalized to ARNT2 expression [58]. In the figures data are shown as mean + standard deviation of the mean (SD).

## Authors' contributions

JVG helped conceive of the study, participated in its design and coordination, carried out the microarray experiments, phylogenetic and synteny analyses, literature review, assisted with the nomenclature, and drafted the manuscript. AMG analyzed the microarray data and performed the statistical analyses for microarray gene expression. AK performed qPCR on unfertilized eggs. JZ, TP, and MJ performed qPCR analyses for confirmation of the microarray results. DRN assisted in the sequence analysis and established the gene nomenclature. JJS conceived and designed the study, obtained support, coordinated its execution, and drafted the manuscript. All authors read, commented on, and approved the final manuscript.

## Supplementary Material

Additional file 1 **Additional Figures**. Orthologous relationships between zebrafish and human CYPs, synteny analyses for human CYP2W1-zebrafish CYP2Ks and human CYP2ABFGST-zebrafish CYP2Ys, expression of control genes, and alternatively microarray expression clustering including CAST results and unnormalized hierarchical clustering. Click here for file

Additional file 2 **Additional Tables**. CYP2J-like genes in zebrafish, CYP genes clustered by developmental expression patterns (CAST), single-color median-scaled Cy3 expression values for microarray probes, and qPCR primer sequences. Click here for file
