# Supplementary material for: Identification and developmental expression of the full complement of Cytochrome P450 genes in Zebrafish
Source: BMC Genomics. 2010 Nov 18;11:643. doi: 10.1186/1471-2164-11-643 (PMC3012610; doi:10.1186/1471-2164-11-643)
Supplement: Additional file 1 — Additional Figures. Orthologous relationships between zebrafish and human CYPs, synteny analyses for human CYP2W1-zebrafish CYP2Ks and human CYP2ABFGST-zebrafish CYP2Ys, expression of control genes, and alternatively microarray expression clustering including CAST results and unnormalized hierarchical clustering. [file 1471-2164-11-643-S1.PDF]

Additional file 1

Title: Supplemental figures

Description: Orthologous relationships between zebrafish and human CYPs, synenty analyses for human CYP2W1-zebrafish CYP2Ks and human CYP2A/B/F/G/S/T-zebrafish CYP2Ys, expression of control genes, and alternatively microarray expression clustering including CAST results and unnormalized hierarchical clustering

Figure S1. Orthologous relationships between zebrafish and human CYP genes

Figure S2. Synteny analysis of zebrafish CYP2K showing relationship to human CYP2W1

Figure S3. Synteny analysis of zebrafish CYP2Y showing relationships to the human CYP2ABFGST cluster

Figure S4. Developmental expression of *beta-actin*, elongation factor 1a (*EF1A*), and *ARNT2* as analyzed by single color microarray

Figure S5. CYP genes clustered by developmental expression patterns

See Additional File 2, Table S2 for genes in each cluster.

Figure S6. Hierarchical clustering of unnormalized CYP gene expression

Figure S1

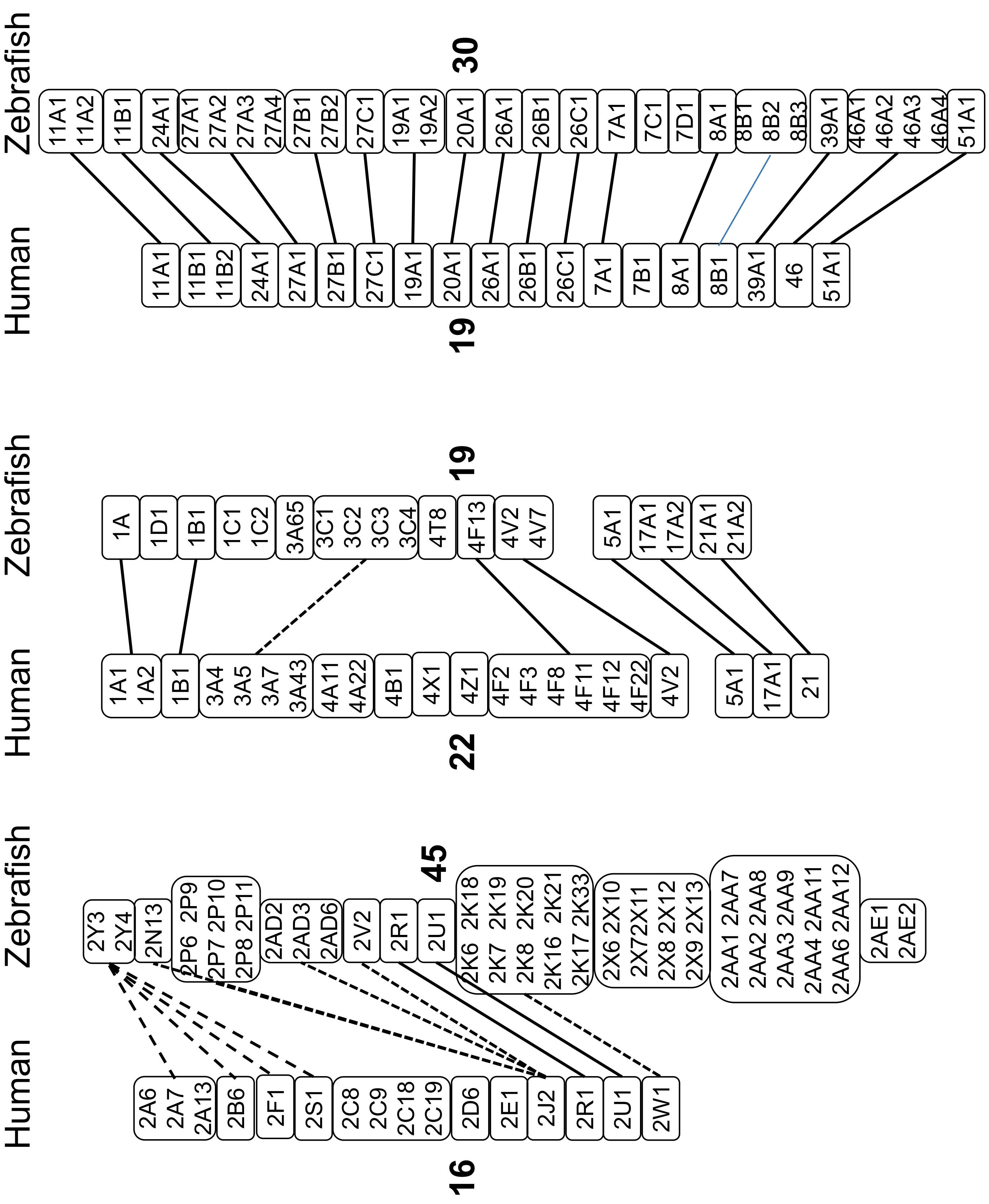

Zebrafish
Human
30
19
11A1 11A2
11B1
24A1
27A1 27A2 27A3 27A4
27B1 27B2
27C1
19A1 19A2
20A1
26A1
26B1
26C1
7A1
7C1
7D1
8A1
8B1 8B2 8B3
39A1
46A1 46A2 46A3 46A4
51A1
11A1
11B1 11B2
24A1
27A1
27B1
27C1
19A1
20A1
26A1
26B1
26C1
7A1
7B1
8A1
8B1
39A1
46
51A1
Zebrafish
Human
19
22
1A
1D1
1B1
1C1 1C2
3A65
3C1 3C2 3C3 3C4
4T8
4F13
4V2 4V7
5A1
17A1 17A2
21A1 21A2
1A1 1A2
1B1
3A4 3A5 3A7 3A43
4A11 4A22
4B1
4X1
4Z1
4F2 4F3 4F8 4F11 4F12 4F22
4V2
5A1
17A1
21
Zebrafish
Human
45
16
2Y3 2Y4
2N13
2P6 2P9 2P7 2P10 2P8 2P11
2AD2 2AD3 2AD6
2V2
2R1
2U1
2K6 2K18 2K7 2K19 2K8 2K20 2K16 2K21 2K17 2K33
2X6 2X10 2X72X11 2X8 2X12 2X9 2X13
2AA1 2AA7 2AA2 2AA8 2AA3 2AA9 2AA4 2AA11 2AA6 2AA12
2AE1 2AE2
2A6 2A7 2A13
2B6
2F1
2S1
2C8 2C9 2C18 2C19
2D6
2E1
2J2
2R1
2U1
2W1

Figure S2

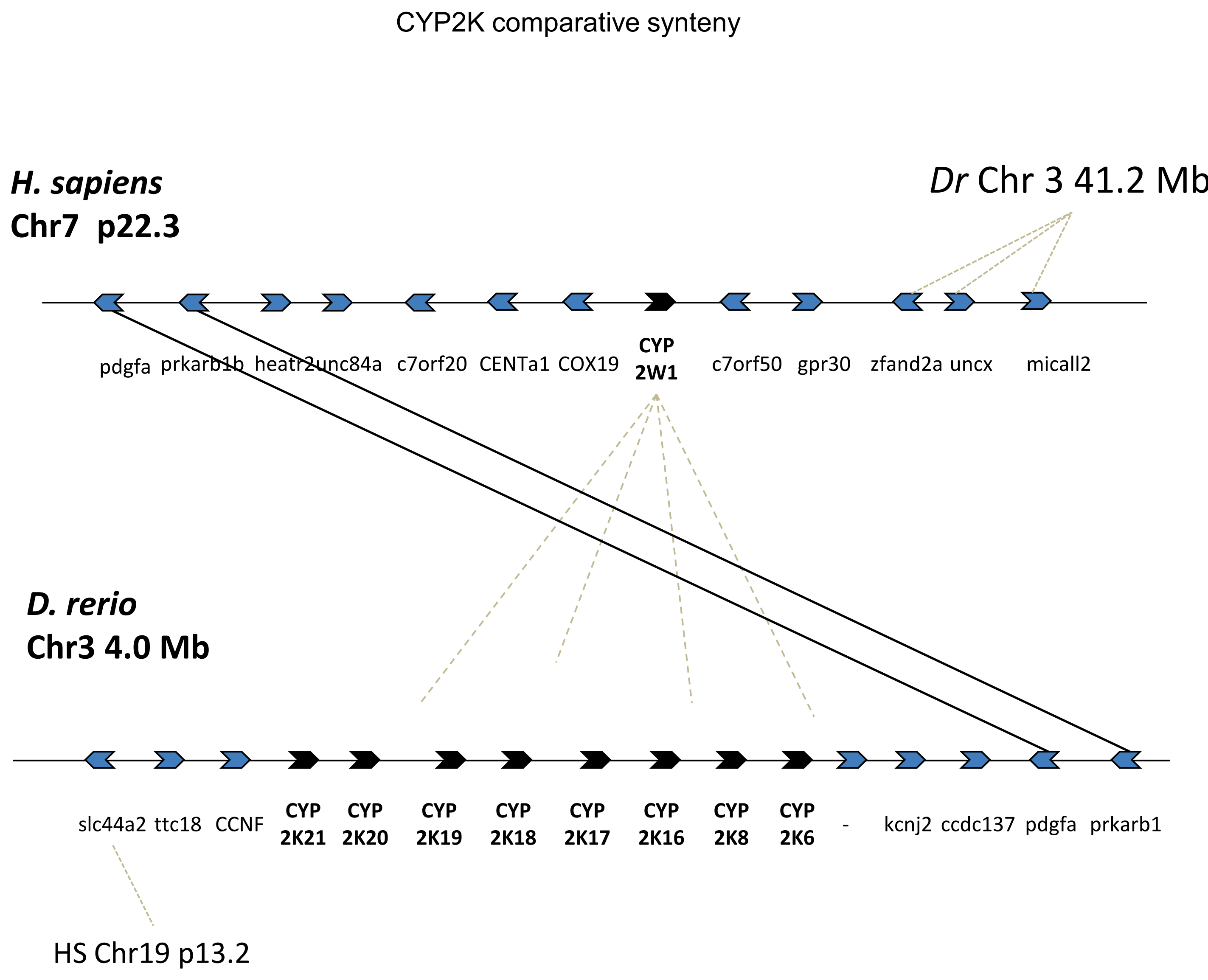
CYP2K comparative synteny
H. sapiens
Chr7 p22.3
Dr Chr 3 41.2 Mb
pdgfa
prkarb1b
heatr2
unc84a
c7orf20
CENTa1
COX19
CYP 2W1
c7orf50
gpr30
zfand2a
uncx
micall2
D. rerio
Chr3 4.0 Mb
slc44a2
ttc18
CCNF
CYP 2K21
CYP 2K20
CYP 2K19
CYP 2K18
CYP 2K17
CYP 2K16
CYP 2K8
CYP 2K6
-
kcnj2
ccdc137
pdgfa
prkarb1
HS Chr19 p13.2

Figure S3

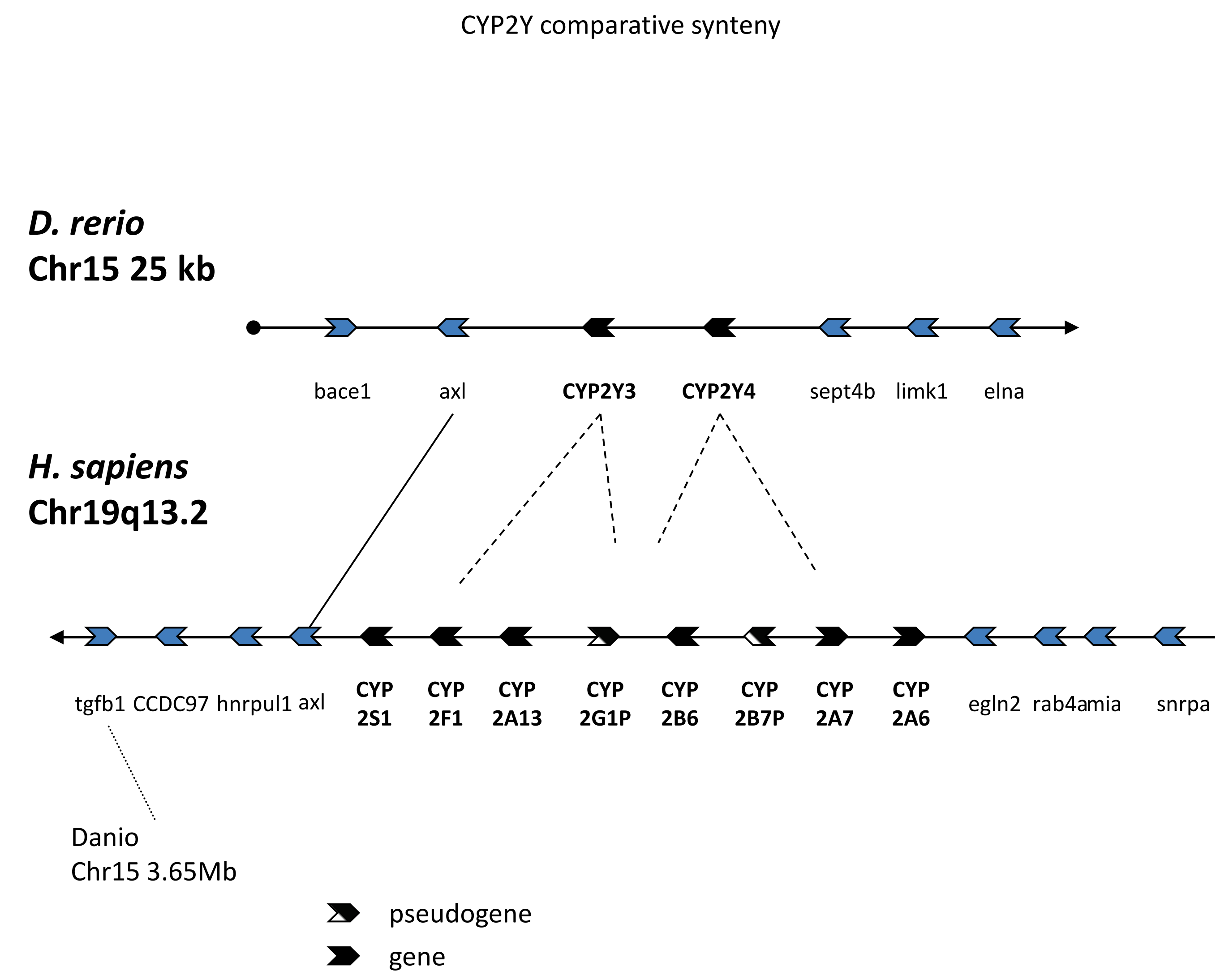
CYP2Y comparative synteny
D. rerio
Chr15 25 kb
bace1
axl
CYP2Y3
CYP2Y4
sept4b
limk1
elna
H. sapiens
Chr19q13.2
tgfb1
CCDC97
hnrpul1
axl
CYP 2S1
CYP 2F1
CYP 2A13
CYP 2G1P
CYP 2B6
CYP 2B7P
CYP 2A7
CYP 2A6
egln2
rab4amia
snrpa
Danio
Chr15 3.65Mb
pseudogene
gene

Figure S4

Single color microarray expression of control genes, including ARNT2, b-ACTIN and EF1A.

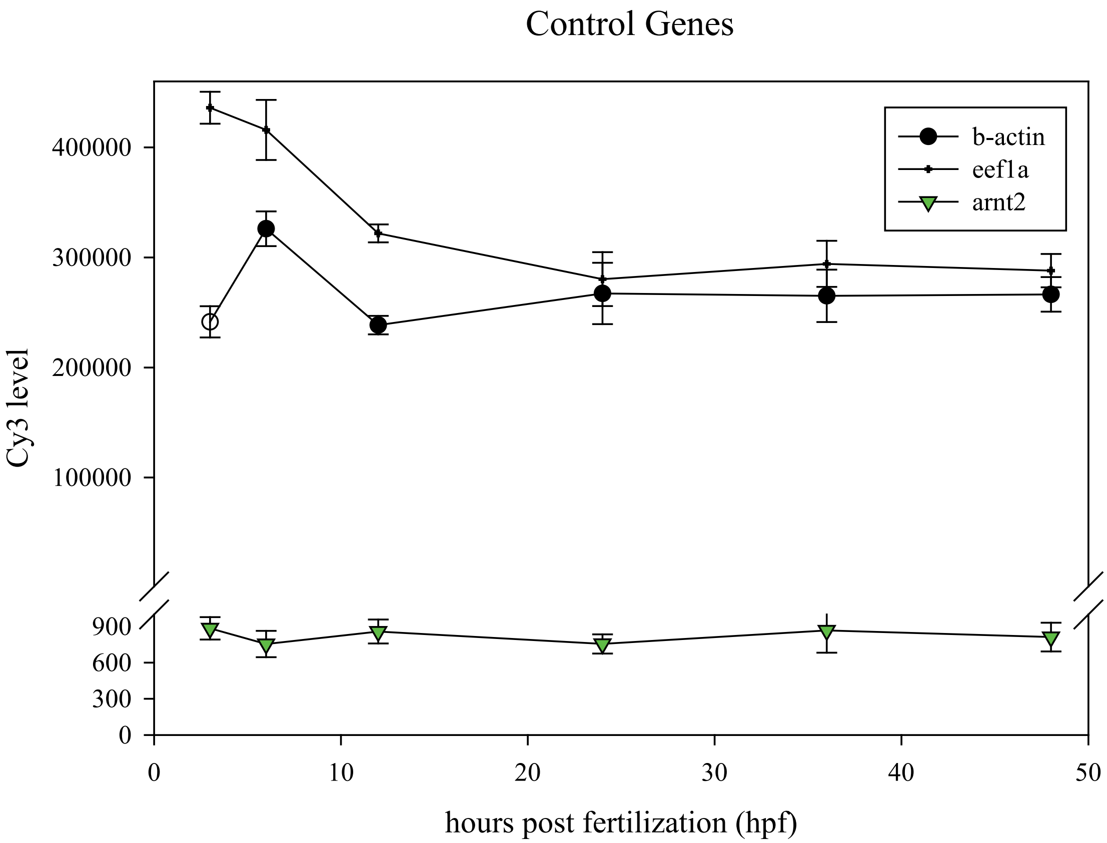

Figure S5

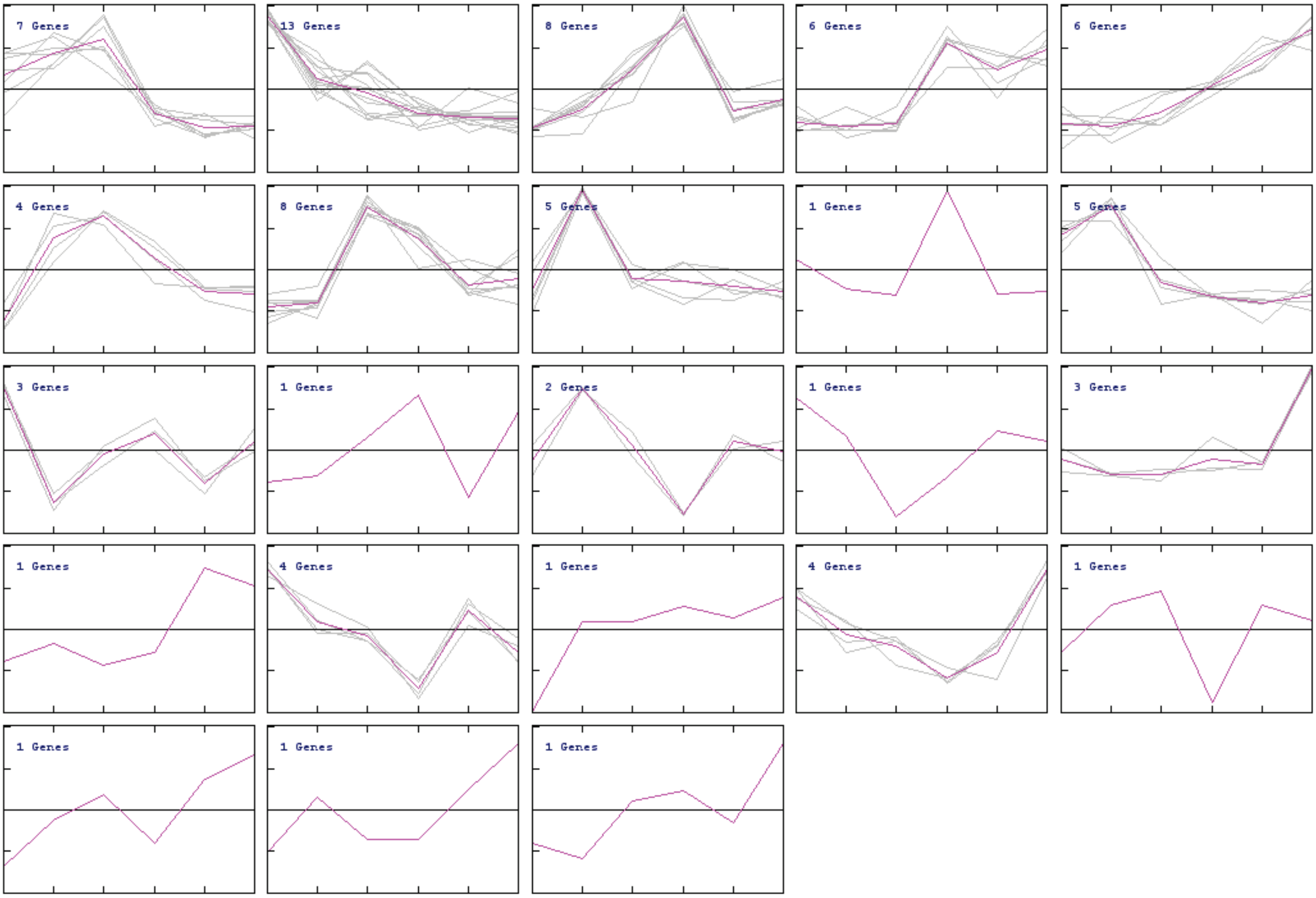
7 Genes
13 Genes
8 Genes
6 Genes
6 Genes
4 Genes
8 Genes
5 Genes
1 Genes
5 Genes
3 Genes
1 Genes
2 Genes
1 Genes
3 Genes
1 Genes
4 Genes
1 Genes
4 Genes
1 Genes
1 Genes
1 Genes
1 Genes

Figure S6

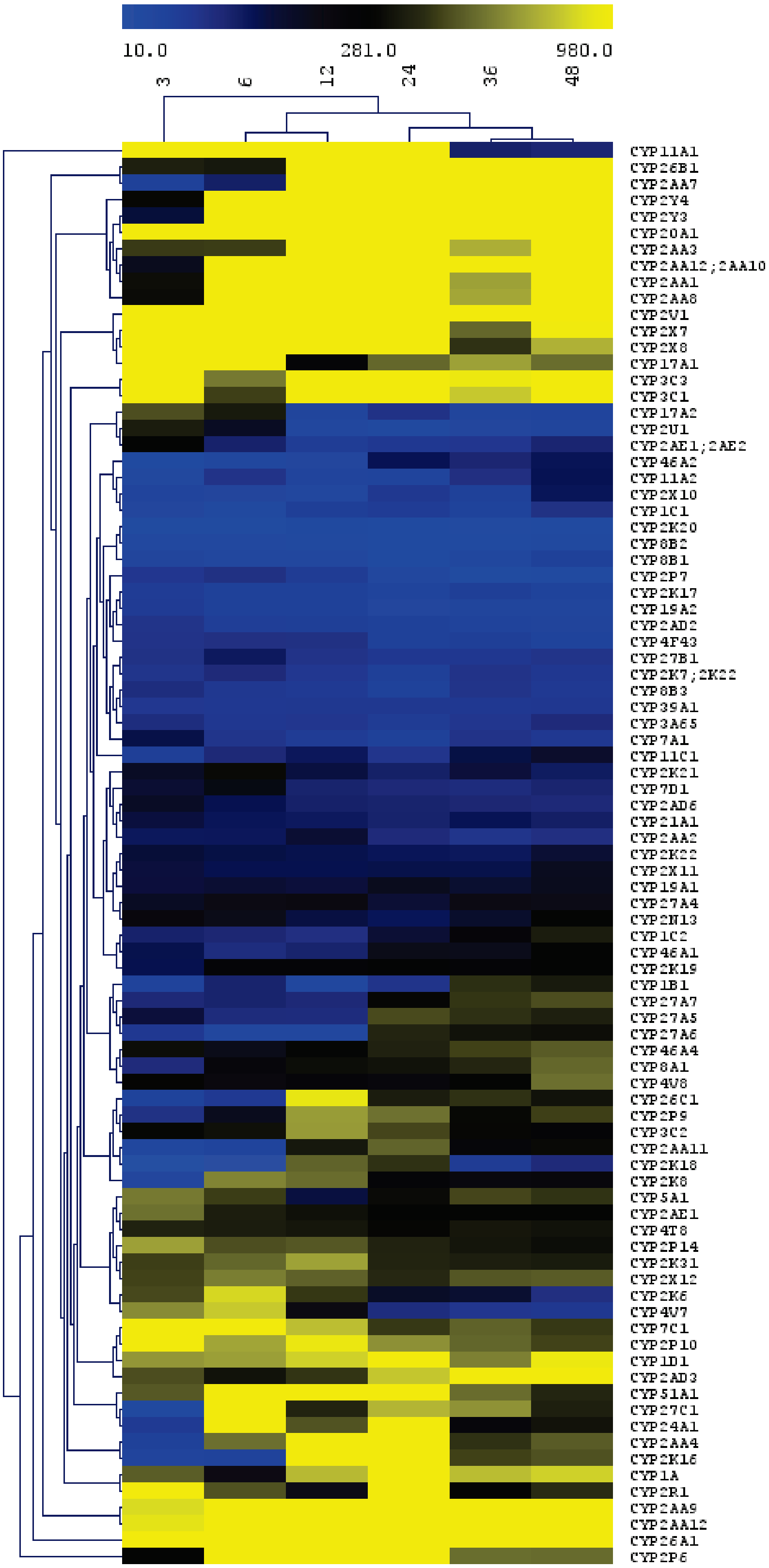
10.0
281.0
980.0
3
6
12
24
36
48
CYP11A1
CYP26B1
CYP2AA7
CYP2Y4
CYP2Y3
CYP20A1
CYP2AA3
CYP2AA12;2AA10
CYP2AA1
CYP2AA8
CYP2V1
CYP2X7
CYP2X8
CYP17A1
CYP3C3
CYP3C1
CYP17A2
CYP2U1
CYP2AE1;2AE2
CYP46A2
CYP11A2
CYP2X10
CYP1C1
CYP2K20
CYP8B2
CYP8B1
CYP2P7
CYP2K17
CYP19A2
CYP2AD2
CYP4F43
CYP27B1
CYP2K7;2K22
CYP8B3
CYP39A1
CYP3A65
CYP7A1
CYP11C1
CYP2K21
CYP7D1
CYP2AD6
CYP21A1
CYP2AA2
CYP2K22
CYP2X11
CYP19A1
CYP27A4
CYP2N13
CYP1C2
CYP46A1
CYP2K19
CYP1B1
CYP27A7
CYP27A5
CYP27A6
CYP46A4
CYP8A1
CYP4V8
CYP26C1
CYP2P9
CYP3C2
CYP2AA11
CYP2K18
CYP2K8
CYP5A1
CYP2AE1
CYP4T8
CYP2P14
CYP2K31
CYP2X12
CYP2K6
CYP4V7
CYP7C1
CYP2P10
CYP1D1
CYP2AD3
CYP51A1
CYP27C1
CYP24A1
CYP2AA4
CYP2K16
CYP1A
CYP2R1
CYP2AA9
CYP2AA12
CYP26A1
CYP2P6
