# Supplementary material for: Identification and developmental expression of the full complement of Cytochrome P450 genes in Zebrafish
Source: BMC Genomics. 2010 Nov 18;11:643. doi: 10.1186/1471-2164-11-643 (PMC3012610; doi:10.1186/1471-2164-11-643)
Supplement: Additional file 2 — Additional Tables. CYP2J-like genes in zebrafish, CYP genes clustered by developmental expression patterns (CAST), single-color median-scaled Cy3 expression values for microarray probes, and qPCR primer sequences. [file 1471-2164-11-643-S2.PDF]

Additional file 2

Title: Supplemental tables

Description: CYP2J-like genes in zebrafish, CYP genes clustered by developmental expression patterns (CAST), single-color median-scaled Cy3 expression values for microarray probes, and qPCR primer sequences.

Table S1. CYP2J-like genes in zebrafish genome

Table S2. CYP genes clustered by developmental expression patterns

See Figure S5 for patterns associated with the gene clusters.

Table S3. Single color median-scaled Cy3 expression values and Agilent probe names for probes used in subsequent analyses

Table S4. qPCR primers used for confirmation of gene expression patterns

Supplemental Table 1. CYP2J-like genes in zebrafish genome.

Gene Name	ENSEMBL ID	ENSEMBL/ZFIN Name	Percentage identity to human CYP2J2
CYP2P9	ENSDARG00000042994	CYP2J20	45.3
CYP2P10	ENSDARG00000042990	CYP2J21	50.5
CYP2P11	ENSDARG00000022631	CYP2J22	49.8
CYP2P8	ENSDARG00000042982	CYP2J23	50.8
CYP2P7	ENSDARG00000042980	CYP2J24	49.7
CYP2P6	ENSDARG00000042978	CYP2J25	46.1
CYP2V1	ENSDARG00000018485	CYP2J26	46.8
CYP2AD3	ENSDARG00000022650	CYP2J27	46.8
CYP2AD2	ENSDARG00000021172	CYP2J28	46.4
CYP2AD6	ENSDARG00000042956	CYP2J29	44.6
CYP2N13	ENSDARG00000042953	CYP2J30	48.4

Supplemental Table 2. CAST analysis of gene clusters. A pattern similarity value of 0.7 was used in the clustering.

Cluster	Genes												
1	CYP2P6	CYP2K31	CYP2AA2	CYP26A1	CYP2X8	CYP4F43	CYP2P7						
2	CYP11A1	CYP2AE1	CYP2AD6	CYP2AD2	CYP2P14	CYP2U1	CYP7C1	CYP19A2	CYP17A2	CYP2P10	CYP2X7	CYP2AE1; 2AE2	CYP7A1
3	CYP2AA9	CYP2AA12	CYP2AA1	CYP2AA12; 2AA10	CYP2AA4	CYP2AA8	CYP2AA11	CYP1A					
4	CYP2AA7	CYP46A2	CYP27A6	CYP27A5	CYP19A1	CYP46A1							
5	CYP26B1	CYP27A7	CYP46A4	CYP2AD3	CYP1C2	CYP8A1							
6	CYP2Y4	CYP2Y3	CYP51A1	CYP2K8									
7	CYP2AA3	CYP2K16	CYP2K18	CYP20A1	CYP3C2	CYP2P9	CYP2K20	CYP26C1					
8	CYP2V1	CYP24A1	CYP27C1	CYP27B1	CYP2K21								
9	CYP2R1												
10	CYP17A1	CYP7D1	CYP4V7	CYP8B2	CYP2K6								
11	CYP3C1	CYP3C3	CYP39A1										
12	CYP1D1												
13	CYP2X12	CYP2K7;2K22											
14	CYP5A1												
15	CYP4V8	CYP2X11	CYP2X10										
16	CYP1B1												
17	CYP4T8	CYP8B3	CYP21A1	CYP2K17									
18	CYP2K19												
19	CYP2N13	CYP8B1	CYP3A65	CYP2K22									
20	CYP27A4												
21	CYP11C1												
22	CYP11A2												
23	CYP1C1												

Supplemental Table S3. Single color median-scaled Cy3 expression values and Agilent probe names for probes used in subsequent analyses

Gene	Probe	3	6	12	24	36	48 hpf
CYP2P6	CUST_84_PI358351581	281	19693	37460	3168	598	589
CYP11A1	CUST_343_PI358351581	33067	10504	9205	3091	132	124
CYP2AA9	CUST_201_PI358351581	888	5224	14865	21267	2570	5403
CYP2AA12	CUST_213_PI358351581	917	5083	15668	20981	3032	5952
CYP26A1	A_15_P110118	11646	18959	16417	8558	3981	4843
CYP2AA7	CUST_212_PI358351581	60	135	2605	11571	5429	8086
CYP26B1	CUST_159_PI358351581	394	366	4897	6447	11335	9846
CYP2AA8	A_15_P106379	324	3106	4854	10320	743	2640
CYP2Y4	A_15_P102911	310	5811	9997	7468	3672	3713
CYP2Y3	CUST_164_PI358351581	175	4932	7050	4994	2559	2373
CYP2AA1	CUST_223_PI358351581	330	1648	3656	6431	728	1502
CYP2AA12;2AA10	CUST_214_PI358351581	213	1166	3421	6085	1299	1721
CYP2AA3	CUST_218_PI358351581	474	481	6000	4178	764	1372
CYP20	CUST_353_PI358351581	2669	2028	5908	5369	3900	3138
CYP2V1	A_15_P114771	1281	5576	2562	1936	1567	1339
CYP2R1	A_15_P111545	1959	527	224	5263	287	439
CYP1A	A_15_P100578	557	229	794	4315	807	862
CYP17A1	A_15_P108178	2548	3510	267	582	728	594
CYP2X7	CUST_179_PI358351581	3359	1363	2331	1248	580	973
CYP2K16	CUST_262_PI358351581	49	52	3211	2407	488	523
CYP2X8	CUST_178_PI358351581	2934	2918	3122	980	455	772
CYP24A1	CUST_183_PI358351581	77	2578	529	980	251	351
CYP2AA4	A_15_P119360	59	600	1181	2367	456	550
CYP3C1	CUST_280_PI358351581	2133	486	997	1462	838	1290
CYP3C3	CUST_275_PI358351581	1901	628	1341	1645	954	1284
CYP27C1	CUST_348_PI358351581	30	1841	412	785	690	394
CYP51	A_15_P106000	543	1665	1813	1175	591	416
CYP2AD3	A_15_P121100	515	347	461	832	1017	1604
CYP7C1	CUST_246_PI358351581	1317	1019	812	468	568	469
CYP2P10	CUST_9_PI358351581	1226	741	947	684	576	490
CYP1D1	CUST_323_PI358351581	701	723	860	1013	648	951
CYP26D1	CUST_111_PI358351581	54	81	931	385	454	349
CYP2K6	A_15_P118829	505	877	466	200	185	105
CYP4V7	CUST_231_PI358351581	673	841	234	108	89	84
CYP2K31	CUST_327_PI358351581	482	578	731	410	392	377
CYP2P14	CUST_66_PI358351581	726	519	540	409	360	333
CYP2P9	CUST_6_PI358351581	100	214	712	609	312	485
CYP3C2	CUST_277_PI358351581	303	336	710	499	306	262
CYP2K8	CUST_190_PI358351581	44	656	592	260	243	250
CYP2X12	CUST_325_PI358351581	490	638	565	428	537	551
CYP5A1	CUST_1_PI358351581	627	479	169	320	500	458
CYP2AE1	CUST_117_PI358351581	607	388	337	284	280	282
CYP4V8	CUST_238_PI358351581	291	242	257	250	280	599
CYP8A1	CUST_134_PI358351581	113	252	333	347	419	581
CYP2AA11	CUST_244_PI358351581	38	50	366	572	256	319
CYP2K18	CUST_240_PI358351581	10	21	571	456	74	116

Supplemental Table S3 continued

Gene	Probe	3	6	12	24	36	48 hpf
CYP46A4	CUST_21_PI358351581	333	219	292	404	487	552
CYP17A2	CUST_315_PI358351581	519	375	46	98	48	50
CYP27A7	CUST_45_PI358351581	118	129	118	297	464	515
CYP27A5	CUST_40_PI358351581	173	110	109	509	453	394
CYP1B1	CUST_335_PI358351581	52	129	38	93	449	375
CYP27A6	CUST_43_PI358351581	82	37	37	414	346	330
CYP4T8	CUST_174_PI358351581	406	382	363	301	364	346
CYP1C2	CUST_284_PI358351581	131	122	104	179	258	383
CYP2U1	CUST_313_PI358351581	381	206	40	32	43	48
CYP2K21	CUST_131_PI358351581	203	317	169	134	173	142
CYP2K19	CUST_150_PI358351581	158	260	260	277	265	288
CYP46A1	CUST_29_PI358351581	159	108	128	220	218	282
CYP2N13	A_15_P121060	245	220	167	151	191	281
CYP27A4	CUST_118_PI358351581	206	230	237	180	230	222
CYP7D1	CUST_36_PI358351581	182	232	128	117	109	126
CYP19A1	CUST_251_PI358351581	172	182	173	214	186	213
CYP2X11	A_15_P120366	171	158	158	161	160	210
CYP2AD6	CUST_19_PI358351581	203	157	132	129	122	117
CYP11C1	CUST_141_PI358351581	65	118	147	91	165	192
CYP2AA2	CUST_298_PI358351581	146	147	182	114	92	104
CYP2K22	A_15_P101602	177	165	159	150	146	181
CYP21A1	A_15_P100466	170	150	143	130	153	136
CYP7A1	CUST_51_PI358351581	163	92	73	62	96	82
CYP11A2	CUST_352_PI358351581	36	96	49	50	104	155
CYP21A2	CUST_346_PI358351581	155	104	99	84	99	89
CYP46A2	CUST_27_PI358351581	29	38	43	154	124	152
CYP2X10	CUST_322_PI358351581	49	45	40	84	60	151
CYP27B1	CUST_15_PI358351581	99	143	96	86	85	95
CYP2K7;2K22	CUST_330_PI358351581	92	115	87	63	96	85
CYP3A65	A_15_P114674	108	85	89	74	89	114
CYP8B3	A_15_P111771	108	84	79	58	94	80
CYP4F43	CUST_147_PI358351581	96	102	101	60	67	53
CYP2P7	A_15_P115152	88	101	74	40	23	27
CYP1C1	CUST_287_PI358351581	41	32	66	72	53	100
CYP2AD2	A_15_P109789	95	68	66	62	52	51
CYP39A1	CUST_91_PI358351581	87	82	84	84	82	85
CYP19A2	CUST_306_PI358351581	75	62	60	43	46	46
CYP2K17	A_15_P117481	73	60	60	50	67	54
CYP8B1	A_15_P107520	47	39	40	29	38	59
CYP8B2	A_15_P119580	34	34	30	29	27	30
CYP2K20	CUST_310_PI358351581	22	23	31	29	24	28

Supplemental Table 4. qPCR primers used for confirmation of gene expression patterns

Gene	Forward	Reverse
Actin	CAACAGAGAGAAGATGACACAGATCA	GTCACACCATCACCAGAGTCCATCAC
ARNT2	CACCTTTGGATCACATCTCATTG	TCACCCTCCTTAGACGGACC
EF1α	CAACCCCAAGGCTCTCAAATC	AGCGACCAAGAGGAGGGTAGGT
CYP1A	GCATTACGATACGTTCGATAAGGAC	GCTCCGAATAGGTCATTGACGAT
CYP1B1	GCTCAGCTGGTCCATTGATACC	CATCAGCGACAGCAACACAC
CYP1C1	AGTGGCACAGTCTACTTTGAGAG	TCGTCCATCAGCACTCAG
CYP1D1	TCAACTTCGACACgaACTGTATC	TGTGAACGATCTGGGAGTTG
CYP2K16	CATGGTGGATCGAGCTAATGAGAG	CTGTCTCCAGTTTTTGAGCAACG
CYP2P6	GAAGGACAACTGAGACATTCCAAGC	GAGATGTGTATCGGAGCTGTGAGG
CYP2R1	GATCAAACGCTTAACATCACCGC	GTCGTGAACAAACCGAGCAAAC
CYP2U1	CAGGTTTGATAGCGTTCGTACTGG	GAGGGATGTTGGCGTATGTCTG
CYP2V1	TATTCAGCTTGCGTCTGGGAAG	CTTGGCACTTGGATATTCTTTCGG
CYP2AA4	TCTTGTTCGCCACTGTTCTCCTAC	TAGCGGACTATACCCACGATGC
CYP2AA12	CCAGGTCATAAAGGAAGCCATAG	CAGTGATCCAGGTTAAAATCGG
CYP11A2	CGGAAAAACCCCGAAGGTAA	GTCCACAGCAGTGTTATAGCCGTC
CYP20	TACAGGAGGTGGAAGGAAAGGTG	GACGACACCAAGGGCATAGATAAC
